# The Molecular Basis for the Calcium-Dependent Slow Afterhyperpolarization in CA1 Hippocampal Pyramidal Neurons

**DOI:** 10.3389/fphys.2021.759707

**Published:** 2021-12-22

**Authors:** Giriraj Sahu, Ray W. Turner

**Affiliations:** ^1^National Institute of Pharmaceutical Education and Research Ahmedabad, Ahmedabad, India; ^2^Department Cell Biology & Anatomy, Cumming School of Medicine, Hotchkiss Brain Institute, University of Calgary, Calgary, AB, Canada

**Keywords:** sAHP, slow AHP, hippocampus, pyramidal cell, KCa3.1, IK, CaV1.3, ryanodine receptor

## Abstract

Neuronal signal transmission depends on the frequency, pattern, and timing of spike output, each of which are shaped by spike afterhyperpolarizations (AHPs). There are classically three post-spike AHPs of increasing duration categorized as fast, medium and slow AHPs that hyperpolarize a cell over a range of 10 ms to 30 s. Intensive early work on CA1 hippocampal pyramidal cells revealed that all three AHPs incorporate activation of calcium-gated potassium channels. The ionic basis for a fAHP was rapidly attributed to the actions of big conductance (BK) and the mAHP to small conductance (SK) or Kv7 potassium channels. In stark contrast, the ionic basis for a prominent slow AHP of up to 30 s duration remained an enigma for over 30 years. Recent advances in pharmacological, molecular, and imaging tools have uncovered the expression of a calcium-gated intermediate conductance potassium channel (IK, KCa3.1) in central neurons that proves to contribute to the slow AHP in CA1 hippocampal pyramidal cells. Together the data show that the sAHP arises in part from a core tripartite complex between Cav1.3 (L-type) calcium channels, ryanodine receptors, and IK channels at endoplasmic reticulum-plasma membrane junctions. Work on the sAHP in CA1 pyramidal neurons has again quickened pace, with identified contributions by both IK channels and the Na-K pump providing answers to several mysteries in the pharmacological properties of the sAHP.

## Introduction

**FIGURE 1:**
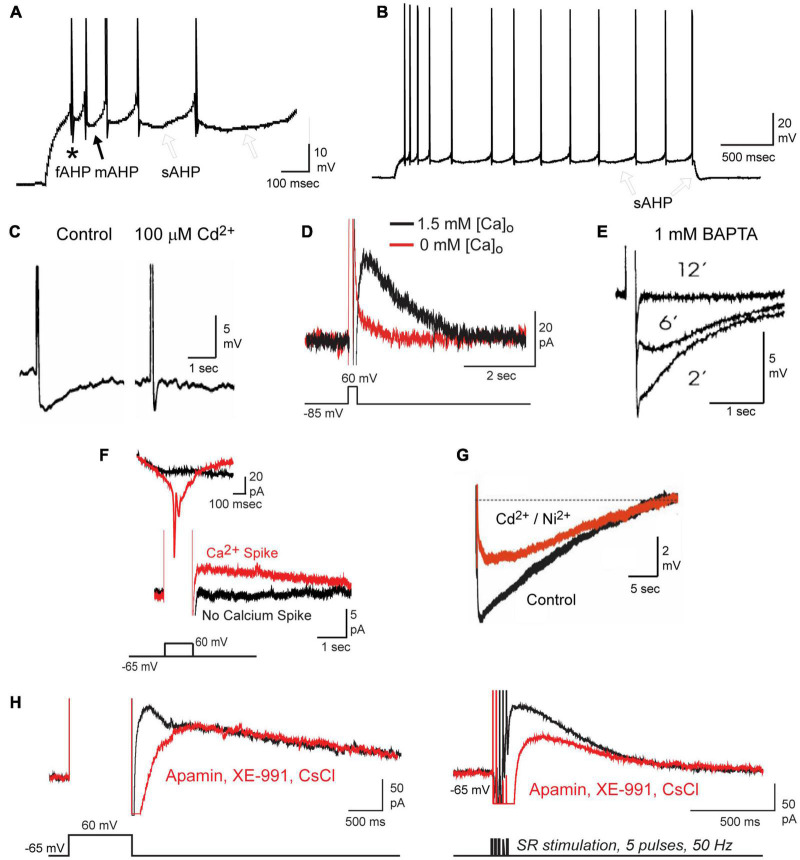
Repetitive spike discharge activates a calcium-dependent sAHP in CA1 pyramidal cells. **(A,B)** Current-evoked spike firing in a CA1 pyramidal cell evokes a sequential series of fAHP (*asterisk*), mAHP (*filled arrow*), and sAHP *(open arrows*) **(A)**. A progressive increase in the sAHP promotes spike accommodation and a post burst sAHP (**B**, *open arrows*). Spikes are truncated in **(A)** for display purposes. **(C–E)** The sAHP is blocked by Cd^2+^ **(C)**, 0 mM calcium medium **(D)**, or over time through internal perfusion of BAPTA **(E)**. **(F)** The IsAHP evoked under voltage clamp by a step depolarization requires activation of membrane calcium conductance, detected here as an unclamped calcium spike. **(G)** The calcium-dependent component of the sAHP evoked by a preceding 50 Hz 150 spike train can be distinguished from an overlapping contribution by the Na-K pump upon perfusion of 200 μM Cd^2+^ and Ni^2+^. **(H)** The IsAHP can be evoked by either a voltage command step or by repetitive synaptic stimulation and recorded in the absence of any contribution by SK (100 nM apamin), Kv7 (10 μM XE-991) or HCN (2 mM CsCl) channels. Figures are modified from King et al. (2015) **(A,B,D,F,H)**, Lancaster and Nicoll (1987) **(C)**, Zhang et al. (1995) **(E)**, and Tiwari et al. (2018) **(G)**. The baseline temperature for each data set was **(A,B,D,F,H)** 32–34°C (King et al., 2015), **(C)** 29–31°C (Lancaster and Nicoll, 1987), **(E)** 34°C (Zhang et al., 1995), and **(G)** 35°C (Tiwari et al., 2018).

Hippocampal CA1 pyramidal cells were one of the first central neurons to draw attention as a model for understanding the factors that control neuronal membrane excitability. The existence of spike-evoked AHPs of different durations were among the first responses reported with intracellular recordings using the *in vitro* slice preparation in the early 1980’s (Alger and Nicoll, 1980; Hotson and Prince, 1980; Gustafsson and Wigström, 1981; Wong and Prince, 1981; Lanthorn et al., 1984; Madison and Nicoll, 1984; Lancaster and Adams, 1986; Lancaster and Nicoll, 1987). Three post-spike AHPs of increasing duration were identified as incorporating calcium-dependent potassium channels: a fast AHP (fAHP, ∼10 ms), medium AHP (mAHP, 50–100 ms), and slow AHP (sAHP, ∼3–20 s) (Figures 1A,B) (for reviews see Storm, 1990; Sah and Davies, 2000; Vogalis et al., 2003b; Stocker, 2004; Adelman et al., 2012; Andrade et al., 2012). Recordings with microelectrodes rapidly established a primary contribution of high voltage-activated calcium currents that activate big conductance (BK, KCa1.1) potassium channels in driving the fAHP and spike repolarization (Lancaster and Nicoll, 1987; Storm, 1987; Shao et al., 1999; Vogalis et al., 2003b; Gu et al., 2007). The mAHP includes contributions by small conductance calcium-dependent potassium channels (SK, KCNN.x) and Kv7 (KCNQ) potassium channels that can influence spike output and synaptic transmission (Storm, 1987, 1989; Gu et al., 2005; Lawrence et al., 2006; Buchanan et al., 2010; Adelman et al., 2012; Chen et al., 2014; Wang et al., 2014; Church et al., 2015). Through years of work the sAHP became recognized as one of the most significant factors controlling spike output in pyramidal cells, and a response that can be realistically considered one of the largest inhibitory responses in the brain. The sAHP was thus shown to be important in controlling synaptic and intrinsic plasticity (Borde et al., 1995, 1999; Sah and Bekkers, 1996; Lancaster et al., 2001; Kumar and Foster, 2004; Le Ray et al., 2004; Fuenzalida et al., 2007; Sametsky et al., 2009; Kaczorowski, 2011; Tedoldi et al., 2020), circuit function with age (Landfield and Pitler, 1984; Campbell et al., 1996; Power et al., 2002; Disterhoft et al., 2004; Tombaugh et al., 2005; Thibault et al., 2007; Matthews et al., 2009; Moore and Murphy, 2020), and if disrupted, leads to repetitive spike output and epileptiform discharge (Alger and Nicoll, 1980; Fernandez de Sevilla et al., 2006; Skov et al., 2009; Tiwari et al., 2019). The sAHP was further distinguished as being under regulatory control by multiple transmitters and second messengers (Madison and Nicoll, 1982, 1986; Lancaster and Nicoll, 1987; Sah and Isaacson, 1995; Pedarzani and Storm, 1996; Zhang et al., 1996; Pedarzani et al., 1998; Haug and Storm, 2000; Lancaster et al., 2001; Melyan et al., 2002; Wong and Schlichter, 2014; Mohan et al., 2019; Tiwari et al., 2019).

Despite identifying several functional roles for the sAHP, defining its underlying molecular basis has been a subject of intense study for over 30 years (Andrade et al., 2012). Recent developments in the pharmacology of potassium channels, super-resolution microscopy, and even a return to microelectrode recordings have renewed the field with multiple findings on the basis for the sAHP. In particular, it has come to light that the sAHP in CA1 pyramidal cells is comprised of one component mediated by calcium-gated potassium channels, and a second component produced by the Na-K ATPase (Na-K pump) that overlaps and extends the calcium-dependent sAHP (Thompson and Prince, 1986; Fukuda and Prince, 1992; Gulledge et al., 2013; Tiwari et al., 2018; Mohan et al., 2019, 2021). Depending on the preceding spike train the calcium-dependent sAHP can extend from ∼3–5 s (10 spikes) up to 20 s (150 spikes), with even more growth of the Na-K phase up to 25–30 s (150 spikes) (Tiwari et al., 2018).

This review will focus on the history of work on two closely related factors: (i) the potassium channels that underlie the calcium-dependent component of the sAHP, and (ii) the calcium sources that drive this response in CA1 pyramidal cells. We thus use the term “slow AHP” primarily in reference to the calcium-dependent component. In this we recognize cell-specific differences in ion channels that can modify or contribute to a slow AHP (i.e., Kv7, Slack, Kir6, HCN, and the Na-K pump), and refer readers to other papers of interest (Schwindt et al., 1989; Maccaferri et al., 1993; Joiner et al., 1998; Sah and Davies, 2000; Faber and Sah, 2003; Wallen et al., 2007; Tzingounis and Nicoll, 2008; Tzingounis et al., 2010; Villalobos and Andrade, 2010; Kaczorowski, 2011; Tanner et al., 2011; Andrade et al., 2012; Gulledge et al., 2013; Chen et al., 2014; Kim et al., 2016; Tiwari et al., 2018; Laker et al., 2021). Given cell-to-cell variability, we largely distinguish between data obtained in CA1 hippocampal pyramidal cells compared to either CA3 pyramidal cells or neocortical pyramidal cells even though there is valuable overlap in some of the findings. To restrict recordings as much as possible to calcium-dependent potassium channels distinct from the mAHP and Na-K pump we focus on the IsAHP typically evoked by a step command or by suprathreshold repetitive spike trains of 5–10 pulses. Using these parameters the typical duration of the calcium-dependent slow AHP and IsAHP is 1–5 s.

The extent of extent of efforts to resolve the molecular basis for the sAHP make it impossible to be all-inclusive in citing examples of key findings in previous work. Indeed, a literature search using the terms “slow AHP OR sAHP AND hippocampus” since 1980 returns over 9,000 results. For brevity we do not review the extensive data involving transmitter and second messenger regulation of the sAHP, or the mechanisms that underlie an increase in sAHP amplitude with age. Rather, we recognize the sum contribution of many labs and thousands of studies that contributed to resolving the factors that produce the sAHP in CA1 pyramidal cells, and choose examples that are representative or can illustrate the path that led to our current understanding.

## The Molecular Identity of Slow Afterhyperpolarization Channels

The sAHP can be evoked synaptically (Lancaster and Wheal, 1984; Lancaster and Nicoll, 1987; Zhang et al., 1996; Lancaster et al., 2001), during repetitive spike discharge (Madison and Nicoll, 1982, 1984), and following the end of a long spike train (Figures 1C–H; Alger and Nicoll, 1980; Hotson and Prince, 1980; Gustafsson and Wigström, 1981; Wong and Prince, 1981). When examined during repetitive discharge evoked by current injection the sAHP grows with successive spikes in the train to promote spike accommodation (Figure 1B; Madison and Nicoll, 1982, 1984). Under voltage clamp the IsAHP can be evoked and distinguished from that of the ImAHP following a step command as an unclamped outward current (Figure 1H; Lancaster and Adams, 1986; Madison et al., 1987). Unlike the fAHP and mAHP, the sAHP was not affected by classical blockers of potassium channels available in earlier years, including apamin, TEA or 4-AP (Lancaster and Nicoll, 1987; Lancaster et al., 1991). Key factors reported in early studies were a block of the sAHP by the scorpion toxin charybdotoxin (ChTx), β adrenoreceptor agonists, and several neurotransmitter modulators (Madison and Nicoll, 1982, 1986; Haas and Greene, 1984; Madison et al., 1987; Sah and Isaacson, 1995; Pedarzani and Storm, 1996; Zhang et al., 1996; Haug and Storm, 2000). However, pinpointing the isoform(s) of calcium-gated potassium channel responsible for the sAHP proved challenging.

### Kv7 Channels

The voltage-gated Kv7 channels (KCNQ family) first known to generate M current also bind calmodulin (CaM) that can confer sensitivity to internal calcium concentration (Marrion et al., 1991; Wen and Levitan, 2002; Gamper and Shapiro, 2003; Gamper et al., 2005; Chang et al., 2018; Zhuang and Yan, 2020). The effects of calcium-CaM interactions on Kv7 channels are complex and often result in a decrease in channel current, with the exact effects depending on the specific combination of co-expressed isoforms (Marrion et al., 1991; Gamper and Shapiro, 2003; Gamper et al., 2005; Chang et al., 2018; Zhuang and Yan, 2020). Each of the Kv7.2, Kv7.3, and Kv7.5 isoforms are expressed in hippocampus, although have differential contributions to the sAHP in principle output neurons of CA1, CA3, and dentate gyrus (Shah et al., 2002; Pan et al., 2006; Tzingounis and Nicoll, 2008; Tzingounis et al., 2010; Kim et al., 2012, 2016). Kv7 channels were thus shown to contribute to the sAHP in CA3 pyramidal (Tzingounis and Nicoll, 2008; Tzingounis et al., 2010; Kim et al., 2012, 2016) and dentate granule cells (Tzingounis and Nicoll, 2008; Laker et al., 2021). In at least one case the Kv7 channel blocker XE-991 was reported to block up to 33% of the sAHP of CA1 pyramidal cells (Tzingounis and Nicoll, 2008) while other studies reported little to no role for Kv7 channels on the sAHP (Aiken et al., 1995; Gerlach et al., 2004; Gu et al., 2005; Tzingounis et al., 2010). This could reflect the understanding that Kv7 channels have a voltage range for activation outside that required to contribute to the sAHP unless subject to modulation by phosphatidylinositol 4,5-bisphosphate (PiP2) (Zhang et al., 2013; Greene and Hoshi, 2016; Kim et al., 2016). It has also been shown that whole-cell recording conditions can wash out factors needed for normal Kv7 function, requiring the use of perforated patch recordings (Loussouarn et al., 2003; Gamper et al., 2005). Given that calcium-CaM interactions often lead to inhibition of specific Kv7 isoforms that are expressed in hippocampus (Gamper and Shapiro, 2003; Gamper et al., 2005; Zhang et al., 2016), and the need for modulatory factors to detect Kv7 function, this review will focus on potassium channels directly activated by calcium.

### SK1 (KCa2.1) Channels

Several lines of evidence came to suggest a role for SK channels in the sAHP, and particularly that of the SK1 isoform through activation by L-type calcium channels. Supporting data came from fluctuation noise analysis and single channel recordings that returned evidence for a small conductance (2–5 pS) potassium channel (Sah and Isaacson, 1995; Selyanko et al., 1998) that was within the range of SK channel isoforms (Kohler et al., 1996; Hirschberg et al., 1998; Marrion and Tavalin, 1998). A set of eloquent recordings revealed a functional coupling between L-type channels and presumed SK channels within single on-cell patch recordings in pyramidal cells (Marrion and Tavalin, 1998). Immunolabels for the SK1 isoform and L-type calcium channels were colocalized in acutely dissociated pyramidal cells (Bowden et al., 2001). Finally, ensemble averages of evoked L-type calcium channels or SK-like potassium channels created macro currents that recapitulated the time course of the sAHP (Cloues et al., 1997; Bowden et al., 2001; Lima and Marrion, 2007). As a result, findings were interpreted to reflect the activity of SK1 channels triggered by L-type calcium influx with properties that would appear to fit the onset, peak, and duration of the sAHP (Tanabe et al., 1998; Lima and Marrion, 2007). However, the introduction of an SK1 knockout mouse that did not affect the sAHP appeared to set aside the possible role for SK1 channels (Bond et al., 2004).

### IK (KCa3.1) Channels

Recordings to assess the role of calcium-gated potassium channels in generating sAHPs were not just restricted to the hippocampus. This was particularly the case for cells in the enteric nervous system of the gastrointestinal tract that generate an sAHP with remarkably similar properties to that of CA1 pyramidal cells (Kunze et al., 1994; Vogalis et al., 2002a,b, 2003a; Furness et al., 2004; Neylon et al., 2004; Nguyen et al., 2007). Work there identified the role of another member of the KCCN family that generates an intermediate conductance calcium-gated potassium channel (KCNN4, SK4, KCa3.1, IK). These channels are from the same gene family as SK1-3 channels with ∼45% homology in sequence (Ishii et al., 1997; Logsdon et al., 1997; Joiner et al., 2001; Kaczmarek et al., 2017), and were often referred to as an SK4 isoform. As for other members of their family, IK channels are voltage-independent and bind CaM to sense intracellular calcium concentration (Khanna et al., 1999; Joiner et al., 2001; Wong and Schlichter, 2014; Lee and MacKinnon, 2018). However, IK channels exhibit a higher conductance in the range of 20–90 pS compared to ∼10 pS for SK channels, and a unique pharmacological profile that includes apamin insensitivity and specific sites for binding of the blockers TRAM-34, Senicapoc, NS-6180, ChTx and maurotoxin (Ishii et al., 1997; Joiner et al., 1997; Logsdon et al., 1997; Jensen et al., 1998; Wulff et al., 2001, 2007; Visan et al., 2004; Ataga et al., 2008; Strobaek et al., 2013; Kaczmarek et al., 2017; Alexander et al., 2019; Brown et al., 2020).

The advances made for cells in the enteric nervous system were almost transferred to hippocampal neurons when the antimycotic drug clotrimazole that blocked both the sAHP in enteric neurons and expressed IK channels (Ishii et al., 1997; Logsdon et al., 1997; Jensen et al., 1998; Neylon et al., 2004) also blocked the sAHP recorded in dissociated hippocampal cultures (Shah et al., 2001). But the best *in situ* hybridization techniques of the day that first identified IK channels did not detect its expression in the brain (Ishii et al., 1997; Logsdon et al., 1997; Jensen et al., 1998; Joiner et al., 2001). The reason for this is unknown as IK channels are expressed in endothelial and smooth muscle cells of the cerebrovasculature and in microglia (Van Renterghem et al., 1995; Neylon et al., 1999; McNeish et al., 2006; Kaushal et al., 2007; Hannah et al., 2011). Added to this were findings that clotrimazole was relatively non-specific in also blocking calcium current and the SK-mediated mAHP (Shah et al., 2001). Finally, since fluctuation noise analysis suggested that the sAHP was produced by a channel with a conductance of ∼5 pS, there was little reason to suspect an intermediate conductance channel as a contributing factor. Together the data came to support a long-held impression that IK channels (KCNN4) were simply not expressed in central neurons and thus not responsible for generating the sAHP in CA1 pyramidal cells (Sah and Faber, 2002; Vogalis et al., 2003b; Stocker et al., 2004; Adelman et al., 2012).

Yet there were growing reports of at least IK immunolabel in primary sensory neurons (Boettger et al., 2002; Mongan et al., 2005), spinal cord motor neurons (Mongan et al., 2005; Bouhy et al., 2011), and rod photoreceptors (Pelucchi et al., 2008). Engbers et al. (2012) directly tested the expression and function of IK channels in rat cerebellar Purkinje cells. Here parallel fiber input activated an sAHP of ∼400 ms that proved to be insensitive to all classic potassium channel blockers including apamin, TEA, 4-AP, or iberiotoxin, but was blocked by ChTx (Engbers et al., 2012). By this time the chemistry of blockers for IK channels had advanced significantly with the introduction of TRAM-34, a triarylmethane drug derived from clotrimazole that blocks the channel at internal sites with an IC_50_ ∼25 nM in expression systems (Wulff et al., 2000, 2001; Jenkins et al., 2013). Bath applied TRAM-34 (100 nM) rapidly blocked the Purkinje cell sAHP in the slice preparation, with complementary tests identifying the presence of KCa3.1 mRNA, and expression of a calcium-gated potassium channel of ∼36 pS that had a direct association with Cav3.2 (T-type) calcium channels (Engbers et al., 2012). It was subsequently shown that this Cav3-IK interaction also provides a repolarizing conductance in Purkinje cell nodes of Ranvier that secures axonal spike propagation (Gründemann and Clark, 2015).

## IK Channels as a Contributing Factor in the CA1 Pyramidal Cell Slow Afterhyperpolarization

### IK Expression

**FIGURE 2:**
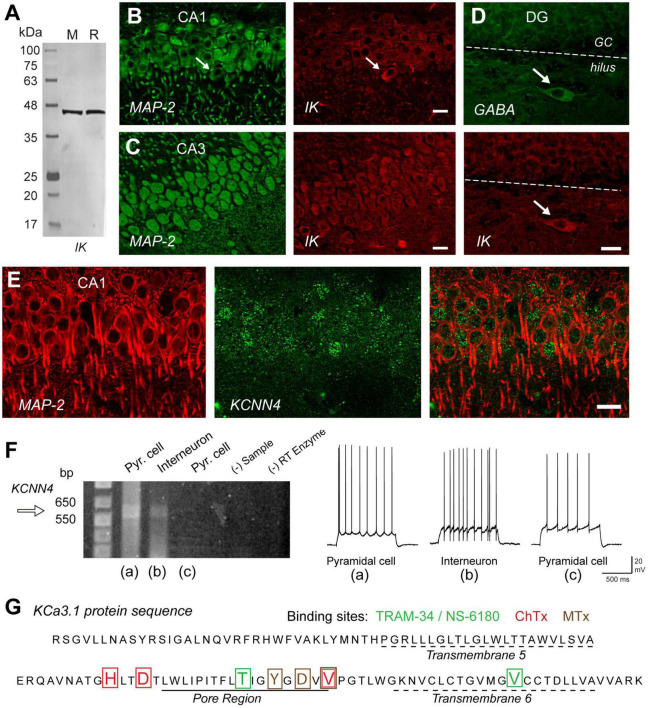
IK channels are expressed in excitatory and inhibitory neurons in the hippocampus. **(A)** Western blot testing the specificity of an IK channel antibody (Santa Cruz D-5, sc-365265) that reports a single band of the correct molecular weight from rat (R) or mouse (M) brain homogenates. **(B,C)** Dual labeling for MAP-2 (*green*) as a structural indicator and anti-IK reveals IK immunolabel (*red*) in pyramidal cell bodies of rat hippocampus in both the CA1 and even higher intensity in CA3. **(D)** Dual immunolabel for GABA and IK in dentate gyrus reveals light IK immunolabel in granule cells and more intense label in a GABAergic hilar interneuron. *Arrows* in **(B,D)** indicate presumed inhibitory cell types with prominent IK immunolabel. **(E)** Dual labeling in CA1 hippocampus for MAP-2 (*red*) and an anti-GFP antibody (*green*) to identify cells expressing GFP in relation to KCa3.1 promoter activity in a transgenic mouse line. **(F)** Single cell RT-PCR of rat KCNN4 mRNA from cells identified electrophysiologically as exhibiting pyramidal cell or interneuron spike patterns before establishing an outside-out seal formation to retain electrode contents. One pyramidal cell and interneuron show a detectable band for KCNN4 cDNA at the predicted band PCR product size. Control lanes lacking sample (- Sample) or reverse transcriptase (RT) enzyme are negative. **(G)** The protein sequence of KCa3.1 translated from pooled samples in **(F)** for single cell RT-PCR cDNA product. The sequence includes transmembrane segments 5 and 6 and the intervening pore region of IK channels, complete with binding sites for four different IK antagonists coded by color to the blocker listed above. ChTx, charybdotoxin; MTx, maurotoxin. Scale bars **(B–E)**, 20 μm. Figures are modified from Turner et al. (2015) **(A–E)** and Turner et al. (2016) **(F,G)**. The baseline temperature for recordings in **(F)** was 32–34°C (King et al., 2015).

Given the evidence for IK expression in Purkinje cells, other brain regions were tested for IK expression using an IK-specific monoclonal antibody and a transgenic mouse line in which GFP expression was tied to promoter activity of the KCNN4 gene (Turner et al., 2015). Control tests confirmed that the antibody labeled a single band on western blots of mouse or rat brain (Figure 2A) and had no cross-reaction with SK channel isoforms. In hippocampus IK immunolabel was detected primarily in the somatic region of neurons with intermediate labeling intensity in CA1 pyramidal cells and even higher intensities in CA3 (Figures 2B,C) and CA4 regions (Turner et al., 2015). IK immunolabel was detected in both pyramidal and GABAergic cells, with notably high levels in dentate hilar interneurons (Figure 2D). These patterns were matched by the pattern of GFP expressed in cells exhibiting KCNN4 promoter activity (Figure 2E). Finally, direct verification of IK mRNA and protein sequence was obtained through single cell RT-PCR from CA1 cells in the rat slice preparation (Turner et al., 2016). Here it was found that cells with spike firing patterns characteristic of pyramidal cells or interneurons exhibited a PCR product size between 550 and 650 bp by using KCNN4-specific primers. This revealed the predicted sequence for IK channel protein surrounding the region of the pore and the presence of binding sites for TRAM-34, NS-6180, ChTx, and MTx. However, the binding site for apamin, the specific blocker for SK channels, was absent from the pore sequence (Figures 2F,G) (Turner et al., 2016).

### IK Channels and Slow Afterhyperpolarization Pharmacology

The collective advances made in defining the expression pattern and pharmacology of IK channels allowed a reexamination of the possibility for IK channels to represent sAHP channels in CA1 pyramidal cells. A series of patch clamp recordings primarily in rat *in vitro* hippocampal slices revealed that the sAHP in CA1 pyramidal cells exhibited the complement of pharmacological properties that define IK channels (King et al., 2015). For these tests all recordings were conducted in the presence of apamin, XE-991, and CsCl to remove any contamination by SK, Kv7 or HCN channel isoforms. The IK channel blocker TRAM-34 was applied at a concentration no higher than 1 μM, a level previously recommended to reduce off target effects (Jenkins et al., 2013). Complementary work also established that this level of TRAM-34 had no effects on BK, Kv7.3, or TMEM16B (Ano2) channels expressed in isolation (King et al., 2015). TRAM-34 was effective in reducing the sAHP with bath application under these conditions. Yet to speed the actions of TRAM-34 in postsynaptic cells while preserving synaptic inputs the majority of recordings were conducted using internal electrode perfusion (King et al., 2015; Turner et al., 2016). This process also enabled the important ability to collect recordings with control electrolyte before adding TRAM-34 to the electrode to achieve a rapid block at its internal binding sites (Wulff et al., 2001; Turner et al., 2016).

**FIGURE 3:**
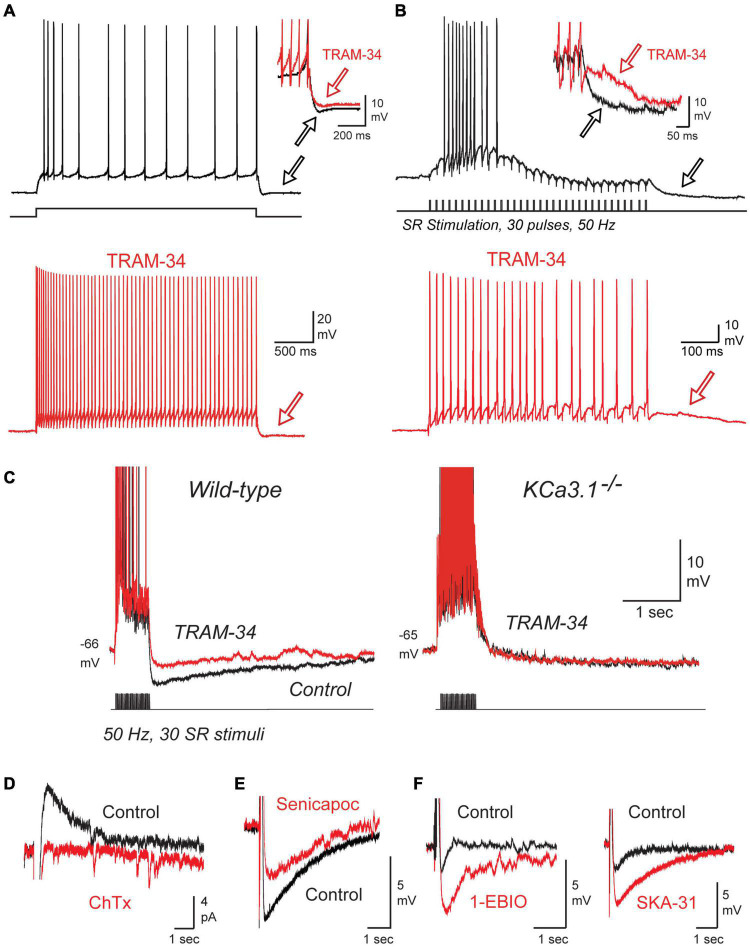
The sAHP and spike accommodation exhibit the unique pharmacological profile of IK. **(A,B)** Repetitive spike discharge in rat CA1 pyramidal cells in tissue slices *in vitro* evoked in response to a square wave current pulse **(A)** or repetitive SR synaptic stimulation **(B)**. Spike accommodation and the post train sAHP (*arrows*) are reduced by 1 μM TRAM-34. **(C)** A current-evoked spike train and afterpotential from CA1 pyramidal cells showing a prominent sAHP in wild type but not KCa3.1^– /–^ mice. TRAM-34 blocks the sAHP in *wt* mice but has no effect on the post-train response in KCa3.1^– /–^ mice. **(D)** The IsAHP evoked by a five pulse 50 Hz SR stimulus train in a perforated patch recording is blocked by local pressure ejection of 100 nM ChTx. **(E,F)** In whole-cell recordings the sAHP evoked after a five pulse 50 Hz SR stimulus train is reduced by the IK blocker Senicapoc (100 nM) **(E)** and enhanced by the IK agonists 1-EBIO (100 μM) or SKA-31 (1 mM) **(F)**. All recordings were conducted in the presence of 100 nM apamin, 10 μM XE-991, and 50 μM picrotoxin to block SK and Kv7 channels and GABA receptors, respectively. Tests on ChTx in **(D)** included 5 mM TEA in the bath to block BK channels. TRAM-34 was internally infused through the electrode in **(A–C)**. Figures are modified from King et al. (2015). The baseline temperature for all recordings was 32–34°C (King et al., 2015).

TRAM-34 was found to block the IsAHP, spike accommodation, and the prominent sAHP that followed repetitive spike trains within 2 min of switching the contents of the electrode from control electrolyte to one containing 1 μM TRAM-34 (Figures 3A,B). Similar tests in mice revealed that TRAM-34 blocked the post burst sAHP evoked following a train of stratum radiatum (SR) inputs in wild type (wt) mice, but had no effect on a low amplitude hyperpolarization (presumably Na-K pump-mediated) in a line of KCa3.1 knockout (KO) mice (Figure 3C; King et al., 2015). To further test specific modulators of IK channels they confirmed that 100 nM ChTx blocked the IsAHP evoked by a brief 50 Hz SR stimulus train (Figure 3D). A block of the SR-evoked post-train sAHP was further obtained in whole-cell recordings with the selective IK channel blocker Senicapoc (100 nM) (Figure 3E; Maezawa et al., 2012). Conversely, the SR-evoked post-train sAHP was increased in amplitude by applying 1-EBIO (100 μM) or SKA-31 (1 mM), two agonists that increase the sensitivity of IK channels to [Ca]i (Figure 3F; Wulff et al., 2007). All together these results built a strong case that sAHP channels in CA1 pyramidal cells exhibit the unique pharmacological profile that defines IK channels (King et al., 2015; Turner et al., 2016).

### Single Channel Recordings

If the sAHP is generated by IK channels then channel properties should be different from those of SK channels, and be evoked in a manner that could explain the long duration sAHP after a preceding spike train. Three studies have used single channel recordings of potassium channels that contribute to the sAHP that have many properties consistent with IK channels.

**FIGURE 4:**
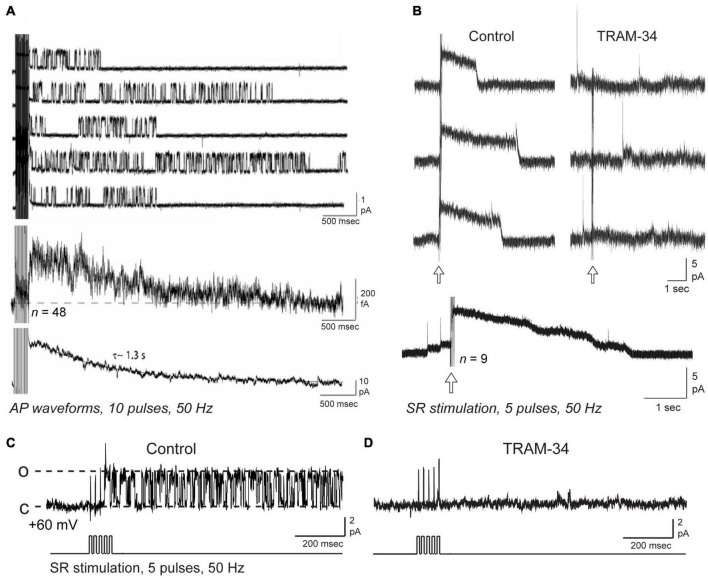
Repetitive synaptic or spike trains trigger IK channel openings over a time course that recapitulates the IsAHP. Shown are on-cell patch recordings of potassium channels from rat CA1 pyramidal cell somata in the slice preparation, with channel current illustrated with respect to the cell interior and open states shown as upward [i.e., *dashed lines* in **(C)** for open (o) and closed (c) states]. **(A)** Somatic on-cell patch recordings of potassium channels using 2.5 mM in the electrode exhibit bouts of channel opening following a train of 10 action potential waveforms. Lowest trace shows the ensemble current generated from 48 null-subtracted sweeps, and for comparison the IsAHP recorded in whole-cell mode from another pyramidal cell. **(B–D)** On-cell patch recordings from CA1 pyramidal cell somata using 3.25 mM potassium in the electrode and a net 60 mV holding potential and pharmacological isolation of IK channels. An outward macroscopic current **(B)** is recorded with a rapid onset and offset following five pulse SR stimulus trains (*arrows*) is blocked by bath applied 1 μM TRAM-34. Lowest trace shows an ensemble average from separate SR stimulus trains. **(C,D)** A single channel (∼36 pS) recorded at the soma shows prolonged bursts of openings following an SR stimulus train **(D)**. Both the macropatch current in **(B)** and SR-evoked single channels **(C,D)** are blocked by bath perfusion of 1 μM TRAM-34. The recordings in **(B–D)** isolated IK channels using 1 μM TTX, 100 nM apamin, 10 μM XE-991, 5 mM TEA, 5 mM 4-AP, and 2 mM CsCl to allow normal activation of synaptic inputs (see King et al., 2015, Supplementary Table S1). Transients in **(B)** reflect capacitive transients from spontaneous spike discharge in the cell. Figures are modified from Lima and Marrion (2007) (Copyright [1997] Society for Neuroscience) **(A)** and King et al. (2015) **(B–D)**. The baseline temperature was **(A)** was 19–24°C (Lima and Marrion, 2007), and **(B,D)** 32–34°C (King et al., 2015).

Lancaster et al. (1991) used inside-out patch recordings in dissociated hippocampal cultured neurons to record calcium-dependent potassium channels that were linear in conductance at hyperpolarized potentials, but exhibited a Mg^2+^-dependent inward rectification for high voltage steps (Lancaster et al., 1991). Thus, under conditions of physiological internal levels of Mg^2+^, a measured value of ∼20 pS for channel conductance at hyperpolarized potentials dropped to ∼10 pS for depolarizing steps. Interestingly, these authors noted that in several cases these “small conductance” channels persisted even in the presence of apamin. Marrion and colleagues used on-cell patch recordings in CA1 pyramidal cells in the rat slice preparation to further identify single potassium channels underlying the sAHP (Marrion and Tavalin, 1998; Bowden et al., 2001; Lima and Marrion, 2007). At the time of their recordings no steps were taken to test for apamin sensitivity. These authors reported a calcium-dependent, voltage-independent channel of 10 pS (Marrion and Tavalin, 1998; Bowden et al., 2001) or ∼19 pS (Lima and Marrion, 2007). They also uncovered an important property where a brief high frequency train (i.e., 10 pulses, 50 Hz) of spike-like command pulses immediately recruited strong bouts of channel openings that could persist for at least 5 s (Figure 4A; Bowden et al., 2001; Lima and Marrion, 2007). Moreover, calculating an ensemble average of these channel openings revealed a current that peaked within 500 ms of the end of the pulse train and decayed with a τ∼1.3–1.6 s, values very similar to the IsAHP evoked under whole-cell conditions with the same form of spike train (Lima and Marrion, 2007).

King et al. (2015) also applied on-cell patch recordings at the soma of rat CA1 pyramidal cells in the slice preparation but under conditions in which IK channel activity was pharmacologically isolated. Using an electrode solution containing 3.25 mM potassium and 1.5 mM calcium at ∼34°C to simulate physiological conditions, recordings revealed a channel of ∼30 pS. These authors also noted an apparent reduction in current amplitude and flickering at high levels of membrane polarization, as previously reported by both Lancaster et al. (1991) and in studies of expressed IK channels (Ishii et al., 1997; Logsdon et al., 1997; Jensen et al., 1998). Again they reported that channel activity was relatively difficult to detect at rest but became immediately evident after a brief, high frequency train of spike-like commands or SR synaptic stimulation (5–20 pulses, 50 Hz). Currents recorded in on-cell or outside-out patch recordings were enhanced by DC-EBIO and blocked by BAPTA-AM or 8-bromo-cAMP (King et al., 2015). Interestingly, SR stimulation uncovered enough channels to evoke a macropatch outward current of 1–5 s duration that exhibited a sharp onset and offset of activity (Figure 4B). Isolated single channels showed the same immediate and long-duration bouts of opening in response to a five pulse, 50 Hz SR stimulus train (Figure 4C). Creating an ensemble average of SR-evoked IK channel activity effectively recapitulated the sAHP as a long duration outward current that decayed over 5 s time (King et al., 2015). Importantly, both single channels and macropatch currents evoked by SR stimulation were blocked by bath application of the IK channel blocker TRAM-34 (Figures 4B,D).

The similarities between the single channel data of Lancaster et al. (1991), Marrion and Tavalin (1998), Bowden et al. (2001), Lima and Marrion (2007), and King et al. (2015) along with those for expressed IK channels (Ishii et al., 1997; Logsdon et al., 1997; Jensen et al., 1998) are striking. While a lower conductance of 10–19 pS was found in first recordings (Lancaster et al., 1991; Marrion and Tavalin, 1998; Bowden et al., 2001; Lima and Marrion, 2007) compared to 30 pS in King et al. (2015), there were differences in recording conditions that could account for this (ion gradients, charge carrier, temperature, Mg^2+^ block, and inward rectification). It is thus very likely that at least some of the first channel recordings of SK-like channel activity and those found under conditions when IK channels are isolated are one and the same.

### IK Channels and the Slow Afterhyperpolarization – Conflicting Results

Work published 1 year after the King et al. (2015) study raised questions as to the role of IK channels given apparent difficulties in detecting IK current or minimal blockade by TRAM-34 applied in the bath or internally through the electrode (Wang et al., 2016). The results appear to contrast entirely with those of King et al. (2015) in reporting no significant effect of TRAM-34 beyond what was attributed to general rundown of current over time. Yet King et al. (2015) had also considered and ruled out the potential influence of run-down of IsAHP currents over 30 min time (see King et al., 2015; Supplementary Figure S3). Some differences between these studies were that King et al. (2015) recorded the IsAHP at near-physiological temperatures vs. room temperature in Wang et al. (2016), although the influence of this on TRAM-34 sensitivity has not been directly addressed. King et al. (2015) also recorded all data in the presence of apamin, XE-991 and CsCl to block SK, Kv7 and HCN channels, whereas these were included in only a subset of recordings in Wang et al. (2016). The potential for this set of blockers to affect recordings remains to be determined. King et al. (2015) further used IK KO mice and recorded a minimal sAHP while Wang et al. (2016) reported a seemingly normal sAHP in IK KO mice that was no different than in *wt* mice. It should be noted that these differences could reflect compensatory mechanisms during development that could include the Na-K pump that have not been fully assessed. Yet both studies agreed in finding that TRAM-34 had no effect on the IsAHP in IK KO mice, a result further supported by a lack of the IK agonist DC-EBIO in IK KO mice (King et al., 2015). Wang et al. (2016) tested TRAM-34 through bath application or in some recordings by including TRAM-34 in the electrode from the outset before obtaining a whole-cell patch configuration. Turner et al. (2016) subsequently clarified the need to use an electrode perfusion system if TRAM-34 is introduced internally given a remarkably fast block of IK channels (1–2 min) if TRAM-34 is present in the electrode upon breaking into whole-cell recording mode. King et al. (2015) used PKAcat or 8-bromo-cAMP to focus on the effects of increasing PKA on the sAHP, recently shown to induce downregulation of IK channels (Tiwari et al., 2019). Wang et al. (2016) used the cholinergic agonist carbachol to increase kinases as a standard test to block and verify the presence of an sAHP with expected properties. The results here might be expected to be different given that carbachol will activate PKA, PKC, and PKG – NO pathways that are now found to also block the Na-K pump-mediated sAHP (Chen et al., 2017; Mohan et al., 2019, 2021; Tiwari et al., 2019). Given the reliance by Wang et al. (2016) on the effects of carbachol to confirm recordings of the sAHP, it is uncertain how much of the response blocked in that study might be attributed to the Na-K pump.

The differences between data sets of King et al. (2015) and Wang et al. (2016) were thus substantial, and revealed at least important effects of the method of applying TRAM-34 (Turner et al., 2016) and the time frame of its effects when applied in the bath or externally. The latter findings are also emphasized by differences between CA1 pyramidal and cerebellar Purkinje cells, in that bath application of even 100 nM TRAM-34 rapidly blocked an IK-mediated sAHP in Purkinje cells (Engbers et al., 2012) compared to a relatively slower block of the CA1 cell sAHP by bath applied 1 μM TRAM-34 (King et al., 2015). Importantly, a block of the sAHP by TRAM-34 has now been repeated in CA1 pyramidal cells and neocortical pyramidal cells (Sahu et al., 2017, 2019; Tiwari et al., 2018, 2019; Roshchin et al., 2020), lending support for the effects of TRAM-34. A specific role for IK channels in pilocarpine-induced epileptic discharge was also reported (Tiwari et al., 2019). The reason for such dramatic differences in the results of the Wang et al. (2016) and King et al. (2015) studies are thus unknown at this time, but suggest some unknown factor(s) that can affect drug sensitivities that remain to be identified.

### IK-SK1 Heteromeric Channel Formation

**FIGURE 5:**
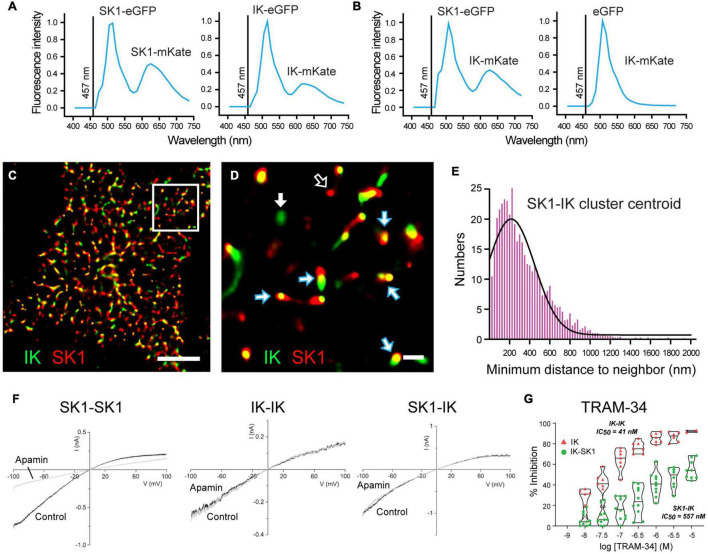
Heteromerization of IK and SK1 channel subunits changes channel properties. **(A,B)** FRET imaging in live tsA-201 cells expressing human SK1 and IK channel subunits as fluorescent constructs to allow eGFP to act as FRET donor and mKate as acceptor molecule upon activation at 457 nm. **(A)** Positive controls confirming that FRET can be used to detect subunit assembly of SK1- or IK-labeled constructs as homomeric channels. FRET is detected as a dual emission for eGFP activated directly by 457 nm excitation, and a second peak for mKate excited by the eGFP emission. **(B)** FRET is elicited if SK1-eGFP and IK-mKate are coexpressed, but not if eGFP alone is coexpressed with IK-mKate. **(C,D)** Super-resolution images using STORM imaging of fluorescent-tagged SK1 (*red*) and IK (*green*) channels expressed in tsA-201 cells. A low resolution image of detected clusters is presented in **(C)** and a magnified view of the Region of Interest in **(D)**. Clusters of fluorescent tags for SK1 and IK are most often overlapping (*blue/white arrows*), or in some cases, present as isolated clusters for IK (GFP, green) (*solid white arrow*) or SK1 (*mKate, red*) (*open white arrow*). **(E)** A right-skewed histogram plot of the nearest-neighbor distances between IK and SK1 clusters reveals a preferential co-association of labels consistent with heteromeric channel formation (bin width, 25 nm). **(F,G)** Membrane currents evoked in outside-out patches from tsA-201 cells expressing human SK1 or IK cDNA in isolation (homomeric assembly) or coexpressing SK1 and IK subunits (heteromeric assembly). Application of 100 nM apamin blocks SK1 homomeric channels but not homomeric IK channels or channels recorded when SK1 and IK subunits are coexpressed **(F)**. Violin plots of the effects of TRAM-34 in tsA-201 cells in **(G)** indicate that bath applied TRAM- 34 blocks homomeric IK channels with IC_50_ = 41 nM but heteromeric SK1-IK channels with an IC_50_ = 557 nM. Scale bars: 5 μm **(C)** and 500 nm **(D)**. Figures are modified from Higham et al. (2019). The baseline temperature for recordings in **(A,B,F,G)** was 22°C (King et al., 2015; Higham et al., 2019).

An explanation for some of the difficulties defining sAHP pharmacology may now have been provided in a study of heteromeric channel formation by IK and SK1 channel isoforms (Higham et al., 2019). This study arose from extensive work on SK channel isoforms showing a species-specific ability to assemble as heteromeric channels, and that heteromerization of potassium channel subunits can change channel properties (Manganas and Trimmer, 2000; Akhtar et al., 2002; Benton et al., 2003; Etxeberria et al., 2004; Monaghan et al., 2004; Sokolov et al., 2007; Al-Sabi et al., 2010; Brueggemann et al., 2011; Church et al., 2015; Autuori et al., 2019). Indeed, it was previously shown that rat SK1 and SK2 subunits can form heteromeric channels in CA1 pyramidal cells that alters apamin sensitivity, while human SK1 and SK2 subunits do not share this property (Church et al., 2015). Marrion and colleagues tested the potential for human SK1 and IK channel isoforms to heteromerize, and any effects this could have on channel activity (Higham et al., 2019). The spatial proximity between these proteins was tested in the tsA-201 cell system to detect fluorescence resonance energy transfer (FRET) between donor and acceptor fluorophores as an indicator of molecules positioned < 10 nm distance. Here FRET was detected between an eGFP donor and mKate acceptor pair for expression of either SK1 or IK subunits, as predicted for subunits that form homomeric channels (Figure 5A). However, FRET was also detected when SK1-eGFP was coexpressed with IK-mKate, revealing a proximity of SK1 and IK subunits that would be expected for a heteromeric channel assembly (Figure 5B). SK1 and IK immunofluorescent labels in tsA-201 cells were further found in close proximity to one another in the membrane through the use of stochastic optical reconstruction microscopy (STORM) (Figures 5C,D). Morphological cluster analysis to quantify nearest neighbor distances between labels revealed a Poisson-like distribution for clusters of a given expressed isoform (i.e., IK-IK, SK1-SK1). However, the histogram for minimal nearest neighbor distances between IK and SK1 clusters was right-skewed, indicating a preferential close association between these subunits (Figure 5E). Together these imaging measures of spatial proximity argue for a prominent level of heteromeric assembly of IK and SK1 subunits when coexpressed.

### IK-SK1 Heteromeric Assembly Alters Channel Properties

Outside-out patch recordings from transfected tsA-201 cells revealed that when expressed in isolation SK1 channels exhibited the expected block by 100 nM apamin, no sensitivity to TRAM-34, and a single channel conductance of ∼20 pS (Figure 5F). IK channels expressed in isolation were apamin-insensitive, fully blocked by TRAM-34 perfusion, and had a single channel conductance of 53 pS (Figure 5F). Yet when IK and SK1 subunits were coexpressed the channel properties changed to being apamin-*insensitive* and with a reduced TRAM-34 sensitivity, shifting from IC_50_ = 21 nm for IK channels expressed in isolation to IC_50_ = 557 nm for coexpressed IK-SK1 subunits (Figures 5F,G). Single channel conductance of IK-SK1 channels also changed to 36 pS, a value midway between the two channels in isolation but in the range expected for an intermediate conductance potassium channel. Finally, a similar decrease in sensitivity was found for ChTx (100 nM) that blocked 95% of homomeric IK channel current but only 30% of IK-SK1 channel current (Higham et al., 2019). Some of the apparent discrepancies in the reported effects of TRAM-34 may then reflect the use of 1 μM TRAM-34 that is closer to the IC_50_ value for heteromeric IK-SK1 channels compared to application at 5 μM (Tiwari et al., 2018). The reduced sensitivity of heteromeric IK-SK1 channels to ChTx might also account for reported difficulties of detecting a block of the sAHP by 10–100 nM ChTx in earlier studies (Lancaster and Nicoll, 1987; Shah and Haylett, 2000, 2002). However, at this time it is not known if these properties apply to native CA1 pyramidal cells in rodents or humans, or if IK channels interact in the same way with SK2 or SK3 isoforms.

### Calcium Sensors

The activation and kinetics of calcium-gated potassium current is also a reflection of the sensitivity of the calcium sensor. One explanation for the slow onset of the sAHP was the potential involvement of an intermediate molecule for its activation. IK channels are known to be gated by CaM that binds to a pocket on the C terminus (Joiner et al., 2001; Wong and Schlichter, 2014) but there is no *a priori* reason to suspect a slow CaM interaction at this site. Growing evidence suggests the role for an additional calcium-sensitive step in hippocalcin, a molecule from a different family of calcium sensors that are expressed in hippocampal neurons (Kobayashi et al., 1992; O’Callaghan et al., 2003). Hippocalcin is normally freely diffusing in a cell until an increase in internal calcium concentration triggers a myristoyl switch at resident EF hands that allows it to translocate to the membrane (O’Callaghan and Burgoyne, 2003; Tzingounis et al., 2007; Andrade et al., 2012). Interestingly, hippocalcin can reversibly translocate to membrane regions in relation to spike-associated activity, and thus potentially respond to calcium increases to modify sAHP amplitude as required (Markova et al., 2008). While several lines of evidence point to a role for hippocalcin, gaining a strict sense of which data apply to CA1 pyramidal cells is difficult since many studies used a combination of dissociated cultured cells, CA3 pyramidal cells or neocortical pyramidal cells *in vitro*.

Potentially the closest comparison is work performed in CA3 pyramidal cells, even though one can expect a greater contribution by Kv7 channels to the sAHP. Thus, it was shown that the IsAHP is substantially reduced in CA3 pyramidal cells of hippocalcin KO mice, where it was also concluded Kv7.3 channels had a major role in generating the sAHP (Kim et al., 2012). The IsAHP was also reduced in hippocalcin KO mice tested in dissociated cells in culture or tissue slices (region unspecified) (Tzingounis et al., 2007). In dissociated rat hippocampal cultured cells the IsAHP was increased by transfecting hippocalcin but not when transfected with a mutant construct that could not be myristoylated (Tzingounis et al., 2007). Similarly, it was found that transfecting hippocalcin in organotypic cultures of neocortical neurons greatly enhanced the IsAHP, while the opposite occurred for transfection of hippocalcin shRNA (Villalobos and Andrade, 2010). The potential influence of more than one calcium sensor protein was indicated when transfection of neurocalcin-δ as an alternate member of this family also increased the IsAHP of neocortical neurons (Villalobos and Andrade, 2010). The site of action of these proteins, however, was not determined.

It is difficult to envision hippocalcin as a calcium sensor in a manner analogous to CaM since an elevation of calcium that triggers myristoylation effectively acts as a switch in function. Relevant here may be reports of hippocalcin acting as an intermediate to AMPA receptor internalization and synaptic plasticity (Palmer et al., 2005; Amici et al., 2009; Dovgan et al., 2010; Jo et al., 2010). Hippocalcin has also been shown to interact with PiP2 (O’Callaghan et al., 2005), leading to the possible transport of PiP2 to the membrane where it could augment Kv7 channel activation (Zhang et al., 2013; Kim et al., 2016, 2017). It is uncertain as to how these data pertain to CA1 pyramidal cells where Kv7 channels have not been recognized as significant contributors to the sAHP. Yet the role for hippocalcin or other members of this family (Haynes et al., 2006; Burgoyne and Haynes, 2012; Raghuram et al., 2012) with regard to the sAHP is a rich target for future work.

### State of the Field

The intensive efforts of hundreds of studies trying to define the molecular identity of sAHP potassium channels have come across unique pharmacological traits that have slowed progress. The reasons for a marked difference between data reported in the King et al. (2015) and Wang et al. (2016) studies remains to be identified. However, in the authors’ view the data for a role for IK channels are at least strong and have been repeated in more than one lab. The recent findings on the outcome of heteromeric combinations of IK and SK channel isoforms serve as another plausible contributing factor to the sAHP that awaits further analysis. The role for alternate calcium sensors opens up a wide range of possibilities for further analysis in the CA1 region.

## Calcium Sources Driving the CA1 Pyramidal Cell Slow Afterhyperpolarization

Equally important to identifying the molecular basis of an AHP are the properties of calcium sources that drive calcium-gated potassium channels. Given difficulties in identifying a direct blocker of “sAHP channels” attention shifted to factors that governed the activation of such a prolonged sAHP.

### Slow Afterhyperpolarization Onset and Decay

A peculiar characteristic of the sAHP is a delayed onset to peak ∼500 ms after a stimulus, and a long rate of decay (τ∼1.5 s) (Jahromi et al., 1999; Gerlach et al., 2004). It was interesting that these properties did not fit the reported fast activation rate of expressed SK channels (Kohler et al., 1996; Hirschberg et al., 1998; Xia et al., 1998). Early studies thus also focused on the calcium sources that drive the sAHP. An initial entry of calcium *via* voltage-gated calcium channels (VGCCs) was established early by block of the sAHP with external Cd^2+^ perfusion (Madison and Nicoll, 1984; Lancaster and Adams, 1986). Introduction through the electrode of EGTA, BAPTA, or other salts with calcium buffering effects reduced or sped the kinetics of the IsAHP (Figure 1E; Madison and Nicoll, 1984; Zhang et al., 1994, 1995; Velumian et al., 1997; Tzingounis et al., 2007). The response of the sAHP was not always as expected, however, where inclusion of low levels of calcium chelators in the electrode could induce a slow increase in amplitude or decay time of the IsAHP during equilibration of electrode contents (Zhang et al., 1995; Velumian and Carlen, 1999). In addition, photolytic activation of either DM-Nitrophen or Nitr-5 to increase internal levels of calcium still evoked an apamin-insensitive outward current with a slow rate of activation to peak 200–300 ms later (Lancaster and Zucker, 1994; Sah and Clements, 1999). All together this led to the understanding of a pattern of relatively slow activation of calcium sources or calcium-dependent potassium channels that delayed the peak of the sAHP, with the duration of the sAHP presumably reflecting the time-course of diffusion/decay of the internal calcium increase (Knöpfel et al., 1990; Müller and Connor, 1991; Lancaster and Zucker, 1994; Sah and Clements, 1999; Gerlach et al., 2004).

### Cav1.3 Calcium Channels

**FIGURE 6:**
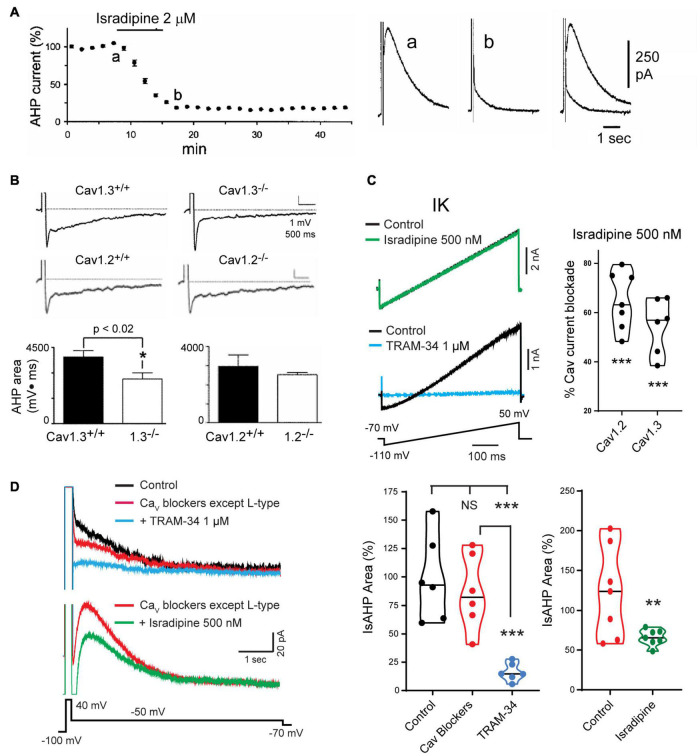
Cav1 L-type calcium channels activate the sAHP. **(A)** The IsAHP is reduced by the L-type channel blocker isradipine. **(B)** The area of the sAHP evoked following a five spike train evoked by current injection is reduced in Cav1.3^– /–^ but not Cav1.2^– /–^ knockout mice. **(C)** Isradipine applied at 500 nM is below a level that affects TRAM-34-sensitive IK channels expressed in tsA-201 cells but provides a substantial block of Cav1.2 and Cav1.3 channel isoforms. **(D)** *Top traces:* The IsAHP in CA1 pyramidal cells with pharmacological isolation of IK channels is not significantly reduced by a suite of calcium channel blockers that excludes dihydropyridines (Cav2.1, ω-Ctx-MVIIC 200 nM; Cav2.2, ω-Ctx-GVIA 1 μM; Cav2.3, SNX-482 200 nM; Cav3.x, TTA-P2 1 μM). Verification of IK channel activation is obtained by a subsequent reduction of the IsAHP by 1 μM TRAM-34. *Bottom traces:* The IsAHP recorded in a separate pyramidal cell in the presence of all calcium channel blockers except L-type is reduced by perfusion of 500 nM isradipine to selectively block Cav3.x channels. Figures are modified from Tanabe et al. (1998) **(A)**, Gamelli et al. (2011) **(B)**, and Sahu et al. (2017) **(C,D)**. **p < 0.01; ***p < 0.001, Student’s paired *T*-test for mean ± SEM. The baseline temperature for recordings was: **(A)** 32°C (King et al., 2015), **(B)** 31°C (Gamelli et al., 2011), **(C)** 22°C (Sahu et al., 2017), and **(D)** 32–34°C (Sahu et al., 2017).

The search for VGCCs that could act as the calcium source established that the IsAHP in intact tissue was not affected by toxins or blockers of N-type (ω-Ctx-GVIA), P/Q-type (ω-Ctx-MVIIC, Aga IVA), T-type (SFTX-3.3, TTA-P2) or R-type (SNX-482) channels (Rascol et al., 1991; Tanabe et al., 1998; Borde et al., 2000; Lima and Marrion, 2007; Kaczorowski, 2011; Sahu et al., 2017). Rather, a role for L-type calcium channel isoforms was shown by a block of the sAHP/IsAHP by verapamil and the dihydropyridines nimodipine, nifedipine, and in particular, isradipine at levels as low as 1–2 μM (Figure 6A; Rascol et al., 1991; Moyer et al., 1992; Lima and Marrion, 2007). A greater specific influence by the Cav1.3 compared to Cav1.2 calcium channel isoform was found in a study of CA1 pyramidal cells of Cav1.x knockout (KO) mice. In these animals the area of the sAHP evoked following a 5 spike train was significantly reduced in Cav1.3 KO but not in Cav1.2 KO mice compared to *wt* mice (Figure 6B; Gamelli et al., 2011). Sahu et al. (2017) revisited this issue to test the role of Cav1 channels capable of activating IK channels in rat CA1 pyramidal cells. This was important in that IK channels were recognized as being highly sensitive to dihydropyridines in the concentration range traditionally used to block Cav1 channels. Thus, IK channels are blocked by dihydropyridines at reportedly low levels of nifedipine (IC_50_ 27 nM), nimodipine (IC_50_ 1 μM), nitrendipine (IC_50_ 27 nM) or verapamil (IC_50_ 28 μM) (Jensen et al., 1998; Wulff et al., 2007).

To selectively block Cav1.x but not IK channels Sahu et al. (2017) tested Cav1 calcium and IK channels expressed in tsA-201 cells. Isradipine proved to significantly reduce IK current at 1 μM or above, but had no effect at 500 nM (Figure 6C; Sahu et al., 2017). By comparison, 500 nM isradipine produced ∼50–60% block of Cav1 current in CA1 pyramidal cells and of Cav1.2 or Cav1.3 expressed in tsA-201 cells (Figure 6C). To test the role of Cav1 calcium channels in activating IK channels they recorded IsAHP in CA1 pyramidal cells with pharmacological isolation of IK channels, and perfused a suite of calcium channel toxins and blockers against low-voltage-activated (LVA) and all high voltage-activated (HVA) channels except Cav1 channels. The IsAHP was not significantly reduced upon perfusion of these calcium channel blockers, but was blocked by subsequent perfusion of 1 μM TRAM-34 (Figure 6D). Repeating this test in the presence of the same LVA/HVA calcium channel blockers followed by perfusion of 500 nM isradipine to selectively target Cav1 channels reduced the IsAHP by ∼40% (Figure 6D). These tests were important in establishing that a selective block of Cav1 calcium channels can reduce the IK-mediated IsAHP in pyramidal cells. Nonetheless, recent work on calcium currents involved in sAHP potentiation suggest that this distinction is not absolute (see below).

### Delayed Facilitation of Cav1 Channels

**FIGURE 7:**
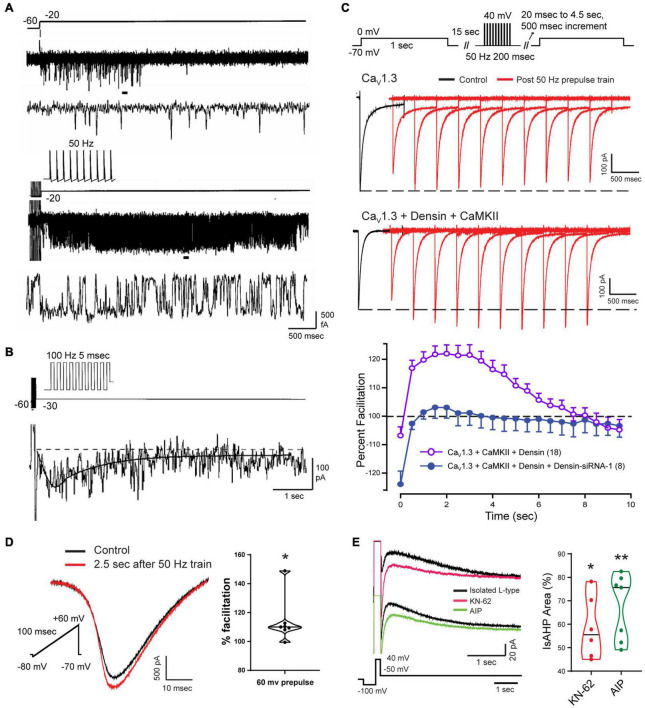
Facilitation of Cav1 calcium current increases the magnitude of the sAHP. **(A,B)** On-cell patch recordings of L-type calcium channels at the soma of CA1 pyramidal cells acutely dissociated from P9-14 rat pups. Calcium channel activity undergoes a delayed facilitation induced by a short train of spike-like responses compared to a 40 mV step depolarization **(A)**. Expanded traces corresponding to horizontal bars show that delayed facilitation reflects an increase in the frequency and duration of openings. Ensemble average (27 sweeps) of L-type channel activity evoked by a 100 Hz train reveals a delayed peak (∼600 ms) and prolonged duration of channel openings (τ∼ 1.6 s) **(B)**. **(C)** Whole-cell recordings from tsA-201 cells subjected to step commands before and after presenting a 50 Hz train of spike-like steps. Cav1.3 channels exhibit a long duration calcium-dependent facilitation (L-CDF) that depends on coexpressing the linking protein densin and αCaMKII. Preincubation of tsA-201 cells with densin siRNA blocks L-CDF of Cav1.3 **(E)**. **(D,E)** Whole-cell recordings in rat CA1 pyramidal cells with L-type current isolated using blockers and toxins against other calcium channels (1 μM ω-conotoxin GVIA, 200 nM Agatoxin, 200 nM SNX-482, and 1 μM TTA-P2). L-type current is facilitated 2.5 s following a 50 Hz train of 200 ms steps to +60 mV **(D)**. The L-type IsAHP is reduced by CaMKII inhibitors KN-62 (10 μM) or AIP (20 μM) **(E)**. Average values in **(C)** are mean ± SEM, with violin plots for data sets in **(D,E)**. *p < 0.05, **p < 0.01; Student’s paired *t*-test. Figures are modified from Cloues et al. (1997) **(A,B)** (Copyright [1997] Society for Neuroscience) and Sahu et al. (2017) **(C–E)**. The baseline temperature for recordings was: **(A,B)** 37°C (Cloues et al., 1997), **(C)** 22°C (Sahu et al., 2017), and **(D,E)** 32-34°C (Sahu et al., 2017).

The activation of VGCCs to promote the calcium influx that generates the sAHP was assumed to take place largely during a preceding spike train or command step. Since the sAHP was sensitive to L-type calcium channel blockers, Cloues et al. (1997) isolated single L-type channels in the on-cell patch mode to test their rate of activation/deactivation (Figure 7). They found that a long step command pulse from –60 to –20 mV evoked a bout of rapid channel openings over a period of ∼500 ms (Figures 7A,B). By comparison, a short 50 Hz train of spike commands activated L-type channels at the end of the stimulus with intense and persistent openings of up to ∼5–6 s (Figure 7B). Ensemble averages of channel openings after a short spike train revealed a delayed facilitation where calcium channel activity rose to an initial peak at ∼500 ms and decayed over a similar time as that of IsAHP. From this and related work it was concluded that the slow activation of sAHP potassium channels reflects at least the properties of the associated L-type calcium channels (Cloues et al., 1997; Marrion and Tavalin, 1998; Bowden et al., 2001). Sahu et al. (2017) later confirmed that the activation of IK channels strongly reflects the voltage-dependence of activation and conductance of either Cav1.2 or Cav1.3 when coexpressed in tsA-201 cells.

### Cav1.x Isoform-Specific Actions

The mechanisms underlying delayed facilitation of Cav1 channel activity are not fully known. However, previous work has revealed Cav1.x isoform-specific properties that could contribute to delayed facilitation. Several studies have reported a transition in L-type channel activity following a depolarizing stimulus to one of long duration openings and high P(o) over time frames of 70–500 ms, although often in the presence of a DHP receptor agonist. Some of the first reports of different gating modes were in cardiac L-type channels (Pietrobon and Hess, 1990; Yue et al., 1990), hippocampal neurons (Fisher et al., 1990; Thibault et al., 1993), cerebellar granule cells (Slesinger and Lansman, 1991, 1996; Forti and Pietrobon, 1993; Koschak et al., 2007), and sensory and motor neurons (Ferroni et al., 1996; Hivert et al., 1999). These properties were reported as a voltage-dependent change in gating pattern (Pietrobon and Hess, 1990; Yue et al., 1990), voltage-dependent potentiation (Kavalali and Plummer, 1994, 1996), or anomalous gating/repolarization openings (Fisher et al., 1990; Slesinger and Lansman, 1991, 1996; Thibault et al., 1993; Koschak et al., 2007). Other work suggested that differential gating reflects two subclasses of channels referred to as Lp (potentiation) vs. Ls (standard), where a stimulus train selectively potentiated the activity of Lp channels beyond the stimulus for up to 200 ms (Kavalali and Plummer, 1994, 1996). Interestingly, the repolarization opening of L-type channels in cerebellar granule cells was traced to the Cav1.2 isoform (Koschak et al., 2007). Cav1.2 channels in ventricular myocytes also exhibit a PKA-dependent increase in membrane expression and formation of clusters through C-terminal linkage to enhance calcium current through cooperative gating interactions (Navedo et al., 2010; Dixon et al., 2012, 2015; Ito et al., 2019a). An analogous process of channel aggregation was reported for a Cav1.3S isoform that has a short C-terminus, but not for a Cav1.3L isoform with a longer C-terminus (Moreno et al., 2016). By forming clusters of 5–8 channels Cav1.3S channels exhibit cooperative gating to increase calcium influx. Yet the majority of studies on these increases in calcium channel activity following a stimulus report an increase in P(o) over a time frame of <500 ms, which is much shorter than that of delayed facilitation that can last ∼6 s (Cloues et al., 1997). Reports of a PKA- or isoproterenol-induced increase in Cav1.2 channel activity is also opposite to that reported of a block of delayed facilitation of Cav1 channel activity by beta adrenergic receptor activation (Cloues et al., 1997).

In CA1 pyramidal cells, a different process supporting CDF was found for the Cav1.3L isoform that contains a PDZ binding domain that interacts with the accessory protein densin. Densin is important as it acts as a bridge to Cav1.3L (but not Cav1.3S) to bind αCaMKII to promote phosphorylation. Jenkins et al. (2010) established in hippocampal neurons that the Cav1.3L-densin-CaMKII interaction increases net calcium current by reducing calcium-dependent inactivation (CDI) of the channel during repetitive stimulation. Sahu et al. (2017) used tsA-201 cells and CA1 pyramidal cells to test for any role of densin in modifying Cav1.3L function following a preceding train of spike-like commands at 50 Hz. They found that when Cav1.3L was coexpressed with densin and αCaMKII in tsA-201 cells it resulted in a long duration calcium-dependent facilitation (L-CDF) of Cav1.3 channel current that could last for up to 8 s (Figure 7C). Moreover, the degree of facilitation increased over the first 500–1000 ms to peak 3–4 s post stimulus by at least 20% over the control test pulse (Figure 7C). As such, the L-CDF enabled by Cav1.3L, densin and αCaMKII effectively recapitulated the time course of an sAHP when coexpressed in tsA-201 cells. However, L-CDF was not evoked in the case of Cav1.3L expressed alone or when coexpressed with only densin or αCaMKII (Figure 7C). By comparison, L-CDF was not observed in tsA-201 cells expressing either Cav1.3S or Cav1.2 coexpressed in combination with densin and/or αCaMKII.

Complementary tests were conducted in CA1 pyramidal cells in the slice preparation using a 50 Hz pulse train followed by a brief test command. These experiments were also carried out in the presence of a suite of calcium channel toxins and blockers to isolate Cav1-mediated calcium current. Here an L-CDF process was detected that amplified Cav1 calcium current by up to 14% when tested 2.5 s after delivering a repetitive stimulus train to 60 mV (Figure 7D; Sahu et al., 2017). Moreover, the IsAHP evoked by a step command was reduced by application of either KN-62 or AIP as CaMKII inhibitors (Figure 7E). This work further supported an earlier report that a CaMKII knockin mouse with deficient autophosphorylation specifically reduced the synaptically evoked sAHP, leading to an increase in CA1 cell excitability (Sametsky et al., 2009).

Together these data suggest that a densin/CaMKII-mediated L-CDF of Cav1.3 channels represents one factor contributing to delayed facilitation that delays the peak and prolongs the duration of an evoked sAHP.

## Ryanodine Receptors

### Functional Coupling of Cav1.3-RyRs

A delayed facilitation of L-type calcium channel activity is able to provide one solution to the long duration sAHP response. However, Cav1.1 and Cav1.2 calcium channels in skeletal and cardiac muscle are known to mediate excitation-contraction (E-C) coupling by linking to ryanodine receptors (RyRs) to enhance intracellular calcium concentration increases (Nakai et al., 1998; Avila et al., 2019). All three RyR isoforms are expressed in CA1 pyramidal cells, including reports of RyR1 and prominent expression of RyR2 and RyR3 (Furuichi et al., 1994; Giannini et al., 1995; Murayama and Ogawa, 1996; Mori et al., 2000; Kim et al., 2007). In other cells a RyR-mediated calcium increase was found to be central to generating the sAHP (Sah and McLachlan, 1991; Jobling et al., 1993; Cordoba-Rodriguez et al., 1999; Pineda et al., 1999; Vogalis et al., 2001). Early work reported variable results in assessing the role for RyRs in generating the sAHP in hippocampal neurons. A RyR-mediated contribution to the sAHP was described for CA3 pyramidal cells in organotypic cultures (Tanabe et al., 1998). In CA1 pyramidal cells some reported that blockers or agonists of RyRs had no effect on the sAHP (Zhang et al., 1995) or only slightly reduced the sAHP in cultured cells (Shah and Haylett, 2000). Yet overall, the data indicate that Cav1 channel isoforms in CA1 and CA3 pyramidal cells are functionally coupled to RyRs to invoke calcium-induced calcium release (CICR) in response to spike activity.

As evidence, calcium imaging in CA1 pyramidal cells established that brief application of caffeine as a RyR agonist evoked large calcium transients up to 600 nM above baseline (Garaschuk et al., 1997; Kim et al., 2007; Berrout and Isokawa, 2009). Caffeine-induced calcium elevations were blocked by higher concentrations of ryanodine or nimodipine, with sensitivity to cyclopiazonic acid (CPA) or thapsigargin to block endoplasmic Ca-ATPases (Garaschuk et al., 1997; Kim et al., 2007; Berrout and Isokawa, 2009). The ability to trigger RyR-mediated calcium increases physiologically came in findings that single or multiple spikes trigger transient elevations in calcium concentration that were sensitive to caffeine, ryanodine, thapsigargin, or CPA (Jacobs and Meyer, 1997; Sandler and Barbara, 1999). Spike-associated increases in internal calcium can also be very focal in that backpropagating spikes produce an L-type channel and RyR-mediated calcium increase localized to dendritic spines (Johenning et al., 2015).

**FIGURE 8:**
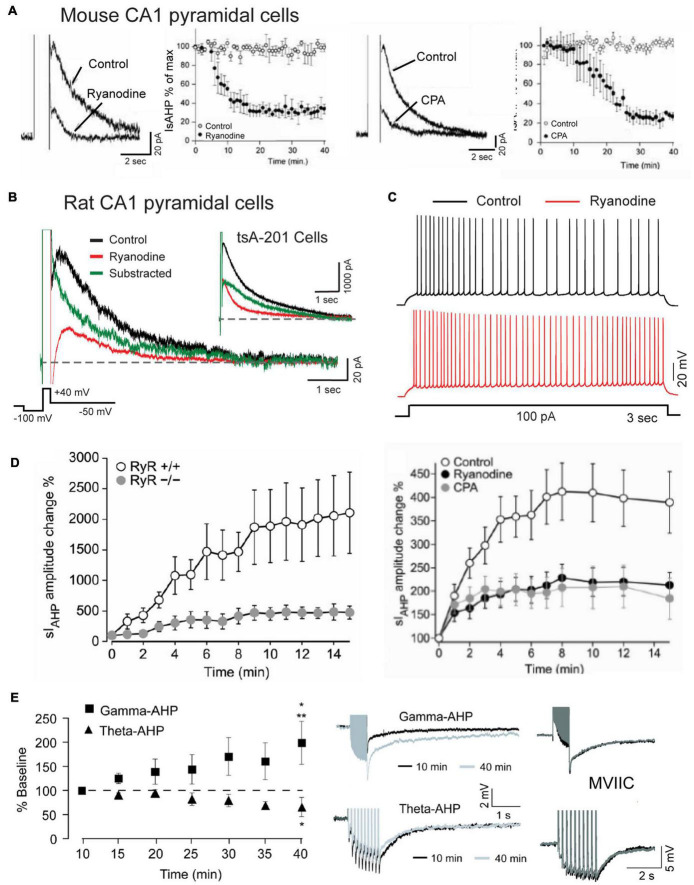
The sAHP is augmented by RyR-mediated calcium release. **(A)** The IsAHP in mouse CA1 pyramidal cells is blocked by ryanodine or the SERCA pump inhibitor CPA. **(B)** Whole-cell recording of isolated IK as an IsAHP in a rat CA1 pyramidal cell before and after infusing 100 μM ryanodine through the electrode. Control and test recordings are superimposed with the difference response. *Inset* shows the same test conducted in tsA-201 cells where coexpressing Cav1.3, IK, and RyR2 produces the same rapid RyR-mediated contribution to an IsAHP-like outward current. **(C)** Spike accommodation in a CA1 pyramidal cell is blocked by 100 μM ryanodine infusion through the electrode. **(D)** A potentiation of the IsAHP in CA1 pyramidal cells with 0.5 Hz activation is blocked in RyR3^– /–^ animals and reduced by pre-perfusion of 10 μM ryanodine or 50 μM CPA. **(E)** sAHP potentiation is selectively evoked by a gamma but not theta frequency pattern of postsynaptic spike firing. Gamma frequency sAHP potentiation is occluded by ω-conotoxin MVIIC (1 μM). Recordings in **(B,C)** were conducted in the presence of 100 nM apamin, 10 μM XE-991 and 2 mM CsCl to block SK, Kv7, and HCN channels, respectively. Average values in **(A,D,E)** are mean ± SEM. Figures are modified from van de Vrede et al. (2007) **(A)**, Sahu et al. (2017) **(B,C)**, Tedoldi et al. (2020) **(D)**, and Kaczorowski (2011) **(E)**. The baseline temperature for recordings was: **(A)** 20–22°C (van de Vrede et al., 2007), **(B)** 32–34°C (Sahu et al., 2017), **(D)** 22°C (Tedoldi et al., 2020), and **(E)** 33–34°C (Kaczorowski, 2011).

Specific involvement of RyRs in generating the sAHP that follows a depolarizing stimulus has been demonstrated in multiple studies where application of higher levels of ryanodine reduced the amplitude and area of the sAHP/IsAHP along with a reduction in spike accommodation (Figures 8A–C; Torres et al., 1996; Borde et al., 2000; Kumar and Foster, 2004; Gant et al., 2006; van de Vrede et al., 2007; Sahu et al., 2019; Tedoldi et al., 2020). This was accompanied by data showing a significant block of the sAHP/IsAHP with effects on spike firing by dantrolene or ruthenium red as alternate blockers of CICR, and by thapsigargin or CPA (Figure 8C; Torres et al., 1996; Borde et al., 2000; Kumar and Foster, 2004; van de Vrede et al., 2007; Tedoldi et al., 2020). Evidence that RyR2-mediated calcium release can activate IK channels was reported by Sahu et al. (2017, 2019) when coexpressing Cav1.3, RyR2 and IK cDNA in tsA-201 cells produced an IsAHP-like response to a step command that is very similar to that of CA1 pyramidal cells (Figure 8B). Calculating the difference between test and control responses of IK recordings further revealed a rapid onset and long duration contribution of the ryanodine-sensitive component in both CA1 pyramidal and tsA-201 cells (Figure 8B; Sahu et al., 2019).

Together these studies established a functional interplay between L-type calcium channels and RyR-mediated CICR in CA1 pyramidal cells that can be triggered by spike-like depolarizations to provide the increase in internal calcium concentration required to activate an sAHP.

### Cav1 Activation of Ryanodine Receptor Isoforms

Evidence has been gained that a block of the sAHP by modulators of CICR may involve specific RyR isoforms. van de Vrede et al. (2007) reported that RyR3 contributes to the sAHP in mouse CA1 pyramidal cells. Here a selective RyR3 antibody infused through the electrode blocked ∼70% of the IsAHP but found no effects when an anti-RyR2 antibody was infused. RyR3 was also implicated in a recent study by Tedoldi et al. (2020), although their results differed in finding that the baseline sAHP in CA1 pyramidal cells in RyR3 KO mice was not significantly different from *wt* animals but still blocked by ryanodine, suggesting a contribution from other RyR isoforms. In the same year Kim et al. (2007) reported that Cav1.3 and RyR2 exhibit direct interactions. Here they used yeast two hybrid and coimmunoprecipitation assays to establish an association at the molecular level between the N-terminus of Cav1.3 (aa 45–115) and N terminus of RyR2 (aa 3150–3680). Interestingly, no coimmunoprecipitation was found between Cav1.2 and RyR2 (Kim et al., 2007), again indicating a more significant role for Cav1.3 compared to Cav1.2 in generating the sAHP. The Cav1.3-RyR2 interaction even extended into increases of RyR2 mRNA by 2 h of depolarization induced by a high K medium, with the mRNA increase reduced by nifedipine or ryanodine, and increased by the L-type agonist BayK 8644.

Remarkably, all aspects of Cav1.3-RyR2 functional coupling in Kim et al. (2007) were reproduced in the absence of extracellular calcium. These results are important in drawing comparisons to the voltage-dependent process found in skeletal muscle through a direct mechanical (non-calcium-dependent) interplay between Cav1.1 and RyR1 (Nakai et al., 1998; Avila et al., 2019). To our knowledge this result stands alone as the only report of a calcium-independent interaction between Cav1.3 and RyR2 or voltage-dependent activation of RyR2 in pyramidal cells. However, the Kim et al. (2007) study was also unique in using a combined high K/BayK 8644 medium to stimulate Cav1.3 channels, which can trigger multiple signaling cascades (Rienecker et al., 2020). The direct association between Cav1.3 and RyR2 established biochemically by Kim et al. (2007) is thus consistent with a role in generating the sAHP, with the proximity of these proteins now verified through FRET imaging (see below) (Sahu et al., 2019). However, it is more difficult to relate the function of a voltage-dependent Cav1.3-RyR2 interaction to generating the sAHP using a High K/Bay K8644 medium, as the initial 3–5 s phase of the sAHP has been shown repeatedly to be calcium-dependent (Figures 1C–G).

### Activity-Dependent Potentiation of the Slow Afterhyperpolarization

An intriguing aspect of the sAHP is an ability to increase or decrease over time in an activity-dependent manner. The first report of this used 2 s current pulses to elicit spike firing presented at long interpulse intervals (Borde et al., 1995). They and others noted a graded reduction in spike firing with pulse intervals as low as 1 per min due to an increase in spike adaptation that was accompanied by an increase in the post train sAHP for up to 170 s (Borde et al., 1995, 2000). The responsible intrinsic factor was calcium-dependent, insensitive to TEA or 4-AP, but blocked by L-type channel blockers and ryanodine, implicating internal calcium release from RyRs as one potential contributor to potentiation (Borde et al., 2000). Indeed, Tedoldi et al. (2020) identified a role for the RyR3 isoform in recordings from a RyR3 KO mouse that revealed a selective reduction in IsAHP potentiation even though the baseline sAHP was not detectably altered (Figure 8D). They also reported that sAHP potentiation with low frequency activation (0.5 Hz) was reduced by either CPA or ryanodine, indicating a complex interplay between calcium release from RyRs and calcium stores, as suggested in control recordings in which the range of variation in degree of potentiation was reflected in values of S.E.M. (Figure 8D).

Kaczorowski (2011) tested the effects of physiologically relevant patterns of spike discharge on the degree of sAHP potentiation. One was designed to mimic a gamma frequency rate of spike discharge using a train of 50 pulses at 50 Hz. The second mimicked a theta burst pattern consisting of 10 bursts of 5 spikes at 100 Hz (interburst frequency 5 Hz). After delivering these stimuli every minute in isolation (or even in alternation) the amplitude of the sAHP was selectively and markedly potentiated by the gamma but not theta frequency train (Figure 8E). Gamma frequency potentiation was further occluded by pre-treatment with nimodipine (10 μM), but curiously, also by ω-conotoxin MVIIC (1 μM) applied as a putative combined N/P-Q type calcium channel blocker (Figure 8E). In addition, gamma frequency sAHP potentiation was blocked by pre-exposure to the HCN channel blocker ZD 7288 (25 μM), a result that was opposite to findings in Borde et al. (1995). However it is worth noting that ZD 7288 has been shown to also block T type calcium current in CA1 pyramidal cells (IC_50_ ∼40 μM) (Sanchez-Alonso et al., 2008). Calcium influx through different channel families might then find a common output by activating RyR3 (Tedoldi et al., 2020), but tests with ryanodine were not included in Kaczorowski (2011).

Intrinsic factors that support potentiation of the sAHP can also affect synaptic plasticity. Thus, the depression of intrinsic excitability induced by current-evoked sAHP potentiation was transferred to a reduction in synaptic responsiveness and spike discharge for 4–5 min (Borde et al., 1999). Conversely, low frequency repetitive synaptic stimulation incorporated postsynaptic sAHP potentiation to increase spike adaptation in response to subsequent trains of synaptic input, and the ability for other synaptic inputs to exhibit LTP (Borde et al., 1999; Le Ray et al., 2004). An early report of an augmented form of NMDA-independent (but nimodipine-sensitive) LTP of synaptic transmission in CA1 pyramidal cells of RyR3 KO mice may then reflect a reduction in postsynaptic IsAHP potentiation, although this was not tested (Futatsugi et al., 1999).

Together these results reveal that sAHP potentiation can be evoked entirely by intrinsic postsynaptic mechanisms that can be selectively recruited according to specific patterns of physiologically relevant spike discharge. They also suggest that calcium channel subtypes beyond L-type calcium channels mediate a gamma frequency-selective sAHP potentiation that can contribute to synaptic plasticity.

## A Cav1-RyR-IK Tripartite Complex Drives the Slow Afterhyperpolarization

**FIGURE 9:**
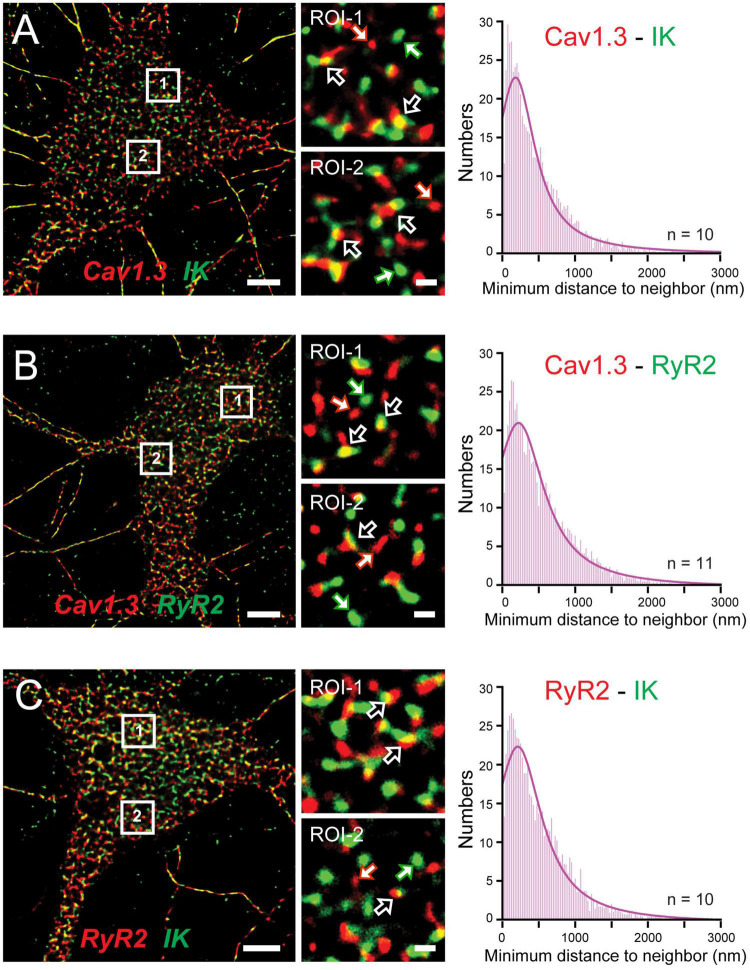
A Cav1.3-RyR2-IK tripartate complex in hippocampal neurons. **(A–C)** dSTORM-TIRF images of dual immunofluorescent labeling in cultured hippocampal neurons for Cav1.3 and IK channels **(A)**, Cav1.3 and RyR2 **(B)**, and RyR2 and IK **(C)**. Representative ROIs identified by numbered boxes enlarged at center reveal clusters of fluorescent tags that present with overlap (*open arrows*) or as isolated signals (*solid white/colored arrows*). At right are histogram plots of the average minimal distance between nearest-neighbor cluster centroids for the indicated pair of labels (bin width, 25 nm). Scale bars: **(A–C)** 5 μm, (**A–C**, ROIs) 500 nm. Figures are modified from Sahu et al. (2019).

The functional coupling that had become apparent between calcium sources and the sAHP led Sahu et al. (2019) to more closely examine the spatial relationship between Cav1.x, RyR2, and IK channels using STORM super-resolution microscopy in dissociated hippocampal neurons (Figure 9). By adding TIRF illumination they could further restrict fluorescent images to within 150 nm of the coverslip surface to focus on near membrane-associated labels. Dual color dSTORM-TIRF imaging found that ∼80–85% of clusters of Cav1.3, RyR2, and IK immunolabels exhibited overlapping emissions (Figures 9A–C). Calculations of the minimal distance to nearest neighbor cluster centroids confirmed close positioning between each of Cav1.3-IK, RyR2-IK, and Cav1.3-RyR2. Expression of eGFP- or mKate-tagged constructs in tsA-201 cells further revealed FRET between each of these pairs of labeled proteins, indicating an association of < 10 nm distance from one another (Sahu et al., 2019). Together these data support the existence of a tripartite complex of Cav1.3-RyR2-IK channels that are optimally positioned to allow calcium-gated activation of potassium channels underlying the sAHP.

Some support was gained for Cav1.2 to also form a similar complex with RyR2 and IK channels. Here morphological cluster analysis reported an even higher density of Cav1.2 than Cav1.3 immunolabel clusters in hippocampal cell membranes (Sahu et al., 2019), as earlier reported for channel expression levels in hippocampal pyramidal neurons (Hell et al., 1993). Yet Cav1.3 and IK immunolabel clusters exhibited overlap almost twice as often as for Cav1.2 and IK clusters. Cav1.3 channels are thus implicated again as a primary membrane-associated calcium source, although a role for Cav1.2 within a Cav1.2-RyR2-IK complex can not be ruled out. The potential role for RyR3 receptors to participate in these complexes was not tested.

### Junctophilin 3/4 Proteins Link the Cav1-RyR-IK Complex at Endoplasmic Reticulum-Plasma Membrane Junctions

**FIGURE 10:**
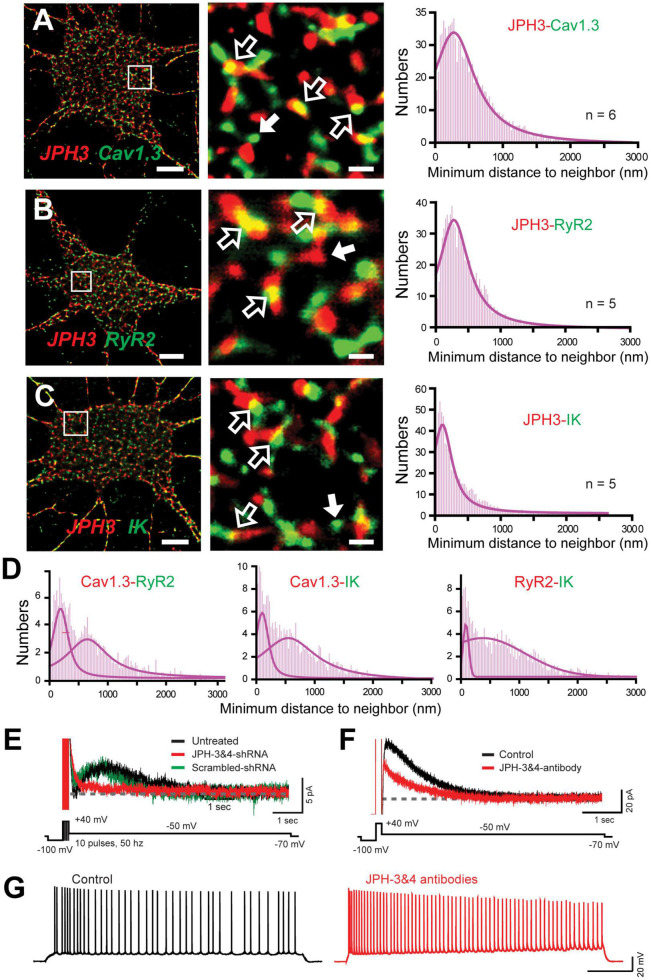
The Cav1.3-RyR2-IK Complex colocalizes with the ER-PM linking protein JPH3. **(A–C)** dSTORM-TIRF images of dual immunofluorescence labeling in cultured hippocampal neurons for JPH3 and Cav1.3 **(A)**, JPH3 and RyR2 **(B)**, and JPH3 and IK **(C)**. ROIs indicated by boxes are enlarged at center, revealing clusters of JPH3 overlap with Cav1.3, RyR2, and IK (*open arrows*). Cases of individual clusters are shown by *solid white arrows*. At right are histogram plots of the average minimal distance between nearest-neighbor cluster centroids for the indicated pair of labels (bin width, 25 nm). **(D)** Minimal distance to neighbor plots from another experiment in which hippocampal cultures were pre-treated with JPH3&4 shRNA for 72 h. Dual dSTORM-TIRF images identified immunolabel clusters for each of Cav1.3, RyR2, and IK proteins. Histogram plots of minimal distance between the indicated protein pairs reveal a bimodal histogram reflecting less overlap of immunolabels, indicating dissociation of the complex without JPH3/4. **(E)** Whole-cell recordings from cultured hippocampal cells of the IK-mediated IsAHP evoked by a train of 10 pulses. Superimposed recordings are from separate cells either untreated or exposed for 72 h to intact or scrambled JPH3 and JPH4 shRNA. Knocking down JPH3/4 blocks the IsAHP. **(F)** Whole-cell recordings of the CA1 pyramidal cell of IK-mediated IsAHP in the slice preparation. The IsAHP is blocked within 10 min of internal infusion of anti-JPH3 and JPH4 antibodies (1:200) through the electrode. **(G)** Whole-cell recordings from CA1 pyramidal cells shows that spike accommodation is blocked within 10 min of infusing anti-JPH3/4 antibodies through the electrode. All recordings in **(A–D)** were conducted in the presence of 100 nM apamin and 10 μM XE-991 to isolate IK channels. Scale bars: **(A–C)** 5 μm, (**A–C**, ROIs) 500 nm. Figures are modified from Sahu et al. (2019), with recordings in **(E)** at 22°C (Sahu et al., 2019).

A striking aspect of a complex of this nature is that it draws on a Cav1.x calcium source expressed at the plasma membrane (PM) and RyRs positioned in the endoplasmic reticulum (ER). It is known from muscle physiology that subunits of Cav1-RyR complexes are maintained in close apposition by Junctophilin 1/2 (JPH1/2) proteins (Piggott and Jin, 2021). In contrast, hippocampal neurons express JPH-3&4 isoforms as one of several classes of linking proteins to help establish ER-PM junctions at distances as close as 30 nm (Nishi et al., 2003; Rowland and Voeltz, 2012; Wu et al., 2017). Indeed, previous studies established that Cav1 and RyR proteins are members of ER-PM junctions, and the mAHP in hippocampal cells is reduced in JPH3/4 KO mice by affecting RyR-SK interactions (Moriguchi et al., 2006; Johnson B. et al., 2018; Vierra et al., 2019). Sahu et al. (2019) used super-resolution imaging to report a close association between JPH-3&4 proteins and subunits of the Cav1.3-RyR2-IK complex (Figures 10A–C). Furthermore, knocking down JPH3/4 expression in cultured cells dissociated the multiprotein complex, as shown by a dramatic reduction in the overlap in cluster immunolabels and a bimodal histogram distribution for nearest neighbor clusters (Figure 10D).

To test the dependence of the sAHP on JPH3/4 protein expression and function, Sahu et al. (2019) conducted whole-cell recordings of the IsAHP in cultured hippocampal neurons and found that pretreatment of cultures with JPH3/4 shRNA for 48–72 h blocked the response, but not in the case of scrambled shRNA (Figure 10E). Similar results were observed in CA1 pyramidal cells in the slice preparation when JPH-3&4 antibodies were infused through the electrode, serving to block the IsAHP (Figure 10F) and spike accommodation mediated by the sAHP in response to current injection (Figure 10G).

## Discussion

**FIGURE 11:**
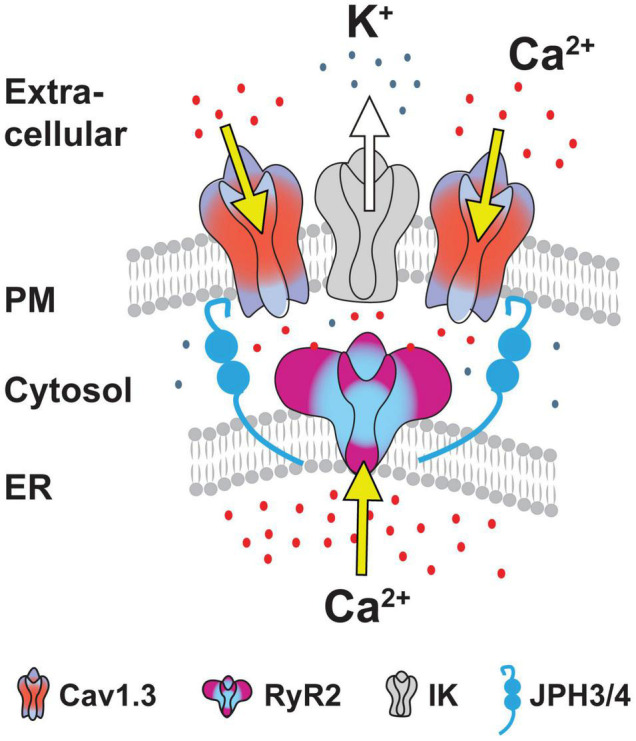
A Cav1-RyR2-IK complex at ER-PM junctions coordinates two calcium sources to activate calcium-gated channels. A cartoon depiction of the tripartate protein complex at ER-PM junctions maintained by at least the linking proteins Junctophilin 3/4. All members of the Cav1-RyR-IK complex exhibit physical proximities at the nanodomain level to enable a tight functional coupling between membrane calcium influx through voltage-gated Cav1.3 calcium channels followed by calcium-induced calcium release by RyRs. A similar tight association with at least IK channels ensures a strong functional coupling between membrane channel voltage and internal calcium concentrations to activation of IK channels as a factor contributing to the sAHP in CA1 pyramidal cells. Figure is modified from Sahu et al. (2019).

The long-sought explanation for the molecular basis for a calcium-dependent sAHP in CA1 pyramidal cells has come closer to being resolved through the combined efforts of hundreds of studies over the past 30 years. The key stumbling blocks in identifying the underlying factors were a perplexing pharmacology and the delayed onset/long duration of the sAHP. Recent work now provides key answers to each of these issues in terms of the activity patterns and pharmacology of both potassium and calcium channels that contribute to the sAHP. In particular, the pharmacology of “sAHP channels” has many parallels to the properties of IK channels and a preferential heteromerization with SK1 channels (King et al., 2015; Turner et al., 2015, 2016; Higham et al., 2019). Secondly, the earlier finding of delayed facilitation of Cav1 calcium channels (Cloues et al., 1997; Bowden et al., 2001; Lima and Marrion, 2007) may reflect at least a densin-CaMKII-mediated interaction with Cav1.3 channels that underlies a novel form of L-CDF (Sahu et al., 2017). A tight functional coupling of IK channels to the activation and conductance of Cav1.3 channels (Sahu et al., 2017) will further ensure a coordinated interplay between these channels (Cloues et al., 1997; Lima and Marrion, 2007; Sahu et al., 2017). Finally, a long-recognized sequential activation of the sAHP by L-type calcium channels and RyR-mediated internal calcium release is explained by the existence of a triprotein complex between Cav1-RyR-IK channels (Figure 11). Proteins in this complex are juxtaposed with nanometer proximity through the actions of JPH3/4 proteins that link subunits at ER-PM junctions that will ensure efficient coupling of both calcium sources to the IK channel (Kim et al., 2007; Sahu et al., 2019; Tedoldi et al., 2020). The accumulated evidence suggests that specific attributes of each of the three members of the complex combine to create a slow onset and long duration calcium-dependent sAHP with unique pharmacological properties.

### Pharmacology of Slow Afterhyperpolarization Potassium Channels

The sAHP in CA1 pyramidal cells defied an onslaught of pharmacological tests over the years to identify the underlying potassium channel isoform. However, advances in defining the pharmacology of IK channels (Wulff et al., 2000, 2007; Kaczmarek et al., 2017) and their expression in CA1 pyramidal cells (King et al., 2015; Turner et al., 2015, 2016) has supported rapid progress over the past 6 years to provide a plausible molecular identity to at least one of the potassium channels that underlie the sAHP. The most recent work obtained in an expression system (Higham et al., 2019) is also pivotal in revealing a strong tendency for human IK and SK1 subunits to form heteromeric channels with characteristics that are remarkably similar to the hallmark identifiers of sAHP channels in CA1 pyramidal cells. Heteromeric IK-SK1 channels exhibit a single channel conductance of 30 pS, and importantly, an altered pharmacology that is insensitive to apamin and an IC_50_ for TRAM-34 of 557 nM – more than 10X higher than that of homomeric IK channels (IC_50_ 41 nM) (Higham et al., 2019). These data could then explain recent differences on the apparent efficacy of TRAM-34 as an IK channel blocker in CA1 pyramidal cells at 1 μM concentration (King et al., 2015; Turner et al., 2016; Wang et al., 2016). Notably, Tiwari et al. (2018, 2019) repeated the tests in CA1 pyramidal cells with bath-applied TRAM-34 at 5 μM, achieving a convincing block of the sAHP with no reported delay. Similar results were reported for Layer 5 neocortical pyramidal cells, where bath-applied TRAM-34 at 5 μM rapidly blocked an sAHP (Roshchin et al., 2020). The use of this higher level of TRAM-34 might then be required to act quickly on an IK-SK1 heteromeric channel population that exhibits a lower drug sensitivity than predicted from studies on homomeric IK channels in expression systems. The validity of this prediction or of any similar interactions with SK2 channels in CA1 pyramidal cells awaits further analysis.

### Cav1 L-type Calcium Channels

Several lines of evidence have built over the years to implicate Cav1 channels, and in particular, the Cav1.3 isoform in generating the sAHP in CA1 pyramidal cells. A key element of calcium channel activity underlying the sAHP is the ability for a short train of high frequency input to invoke a delayed facilitation of Cav1 channel openings. One contributing mechanism to this process is the L-CDF of Cav1.3 (but not Cav1.2) channels that develops through an interaction between the Cav1.3L C-terminus and densin that acts as a bridge to CaMKII. An L-CDF of calcium current was detected in CA1 pyramidal cells, as was the influence of CaMKII on the sAHP and CA1 cell excitability (Sametsky et al., 2009; Sahu et al., 2017).

Despite a direct role for Cav1.3 in mediating L-CDF, there are other ways that Cav1.2 channels could contribute to this process. For instance, while the specific isoform that could account for Lp channels that exhibit late reopening in pyramidal cells was not identified, the Cav1 isoform that exhibits late reopening in dentate gyrus granule cells was traced to Cav1.2 (Koschak et al., 2007). Given that CaMKII has been shown to phosphorylate Cav1.2 channels to increase channel open probability (Yuan and Bers, 1994; Hudmon et al., 2005; Lee et al., 2006; Blaich et al., 2010) it is possible that the Cav1.3L-densin-CaMKII interplay could recruit Cav1.2 channel activity through phosphorylation. We note that PKA phosphorylation can also increase Cav1.2 channel activation (Kavalali et al., 1997; Hall et al., 2007), suggesting the potential for PKA to recruit Cav1.2 channel openings. This seems unlikely, however, given that delayed facilitation of Cav1 calcium channels was blocked by isoproterenol expected to increase PKA (Cloues et al., 1997). Finally, the possibility that an activity-dependent aggregation of Cav1.2 or Cav1.3S channels could contribute to delayed facilitation remains to be tested (Dixon et al., 2012, 2015; Moreno et al., 2016; Ito et al., 2019b). This action could be envisaged if JPH3/4 or other factors provide an activity-dependent change in ER-PM junctions or recruitment of subunits to the complex (de Souza et al., 2015; Johnson B.T. et al., 2018; Kirmiz et al., 2018; Tao-Cheng, 2018; Vierra et al., 2019). In this regard, Kv2 potassium channels have been found to be key factors in controlling Cav1 channel participation in ER-PM junctions that has not been tested yet in the context of the sAHP (Mandikian et al., 2014; Fox et al., 2015; Kirmiz et al., 2018; Johnson et al., 2019; Vierra et al., 2019).

It is also important to note that an equivalent role for Cav1 channels driving IK channels and the sAHP can not be assumed in other cells. For instance, neocortical pyramidal cells in Layers II/III that exhibit distinct firing patterns activate an sAHP with other calcium channel isoforms, and ryanodine blockers have differential effects on these cell types (Pineda et al., 1998, 1999). In cerebellar Purkinje cells IK channels colocalize with Cav3.2 T-type calcium channels to generate an sAHP ∼400 ms duration activated even by low amplitude subthreshold parallel fiber EPSPs (Engbers et al., 2012). The function of the IK channel-mediated sAHP is also different in Purkinje cells in establishing a frequency-dependent control of synaptic input (Engbers et al., 2012) and maintaining spike output at nodes of Ranvier (Gründemann and Clark, 2015). The roles for IK channels in controlling cell excitability in central neurons are thus only beginning to be identified.

## Ryanodine Receptor Contributions to a Cav1-RyR-IK Complex

The extent of linkage between RyRs and Cav1 channels as part of a complex has steadily emerged, with an association detectable using yeast two hybrid analysis, coimmunoprecipitation, immunolabel localization, morphological cluster analysis, and FRET (Kim et al., 2007; Sahu et al., 2019; Vierra et al., 2019). The tight association of RyR2 with IK channels as part of a Cav1-RyR-IK complex that depends on JPH3/4 linking proteins further identifies their location at ER-PM junctions. The recent report of the RyR3 isoform contributing to sAHP potentiation reveals the functional specificity of different RyR isoforms (Tedoldi et al., 2020).

It is interesting to note that the Cav1-RyR association within a complex here has many similarities to the complex formed in cardiac and skeletal muscle for excitation-contraction (E-C) coupling. In skeletal muscle this reflects an association between Cav1.1 and RyR1, while in cardiac tissue it is Cav1.2-RyR2 retained at the interface of membrane and sarcoplasmic reticulum by JPH1/2 (Dirksen, 2002; Piggott and Jin, 2021). This is important, as years of work on E-C coupling have identified multiple levels of interaction between these subunits. In particular, it emerged that Cav1.x and RyRs exhibit a bidirectional interplay, such that calcium- or voltage-dependent Cav1 activation can modify RyR-mediated calcium release in a tissue-specific manner (Avila and Dirksen, 2000; Avila et al., 2001, 2019; Dirksen, 2002; Heck et al., 2021). Indeed, calcium released by RyRs can augment Cav1 channel activity (Mitterdorfer et al., 1996; Nakai et al., 1996; Grabner et al., 1999; Bers and Morotti, 2014), increase Cav1 channel expression levels (Avila et al., 2001), or reduce Cav1 channel conductance by promoting CDI (Bers and Morotti, 2014). Some of these effects in skeletal muscle have been tracked to specific residues on RyR1 that invoke voltage-dependent interactions and enhance Cav1.1 function (Nakai et al., 1998).

In the hippocampus this complex is replaced by at least a Cav1.3-RyR2-JPH3/4 combination (Figure 11). The full extent of interactions between these protein partners or their modulation to regulate the sAHP remains to be determined. Relevant to this may be reports of an interplay between mGluR activation and RyR-mediated augmentation of Cav1 current in cerebellar granule cells (Chavis et al., 1996). Interestingly, these effects appear to be distinct from those which might arise from calcium release by IP3Rs (McPherson et al., 1991; Johenning et al., 2015; Chen-Engerer et al., 2019), but the interactions between synaptic inputs, RyRs, and IP3Rs is complex and cell specific (Fagni et al., 2000; Miyazaki and Ross, 2013). Finally, the targets for phosphorylation by CaMKII associated with Cav1.3-densin have not been determined. This should be central to the entire process of delayed facilitation and L-CDF since CaMKII has been shown capable of phosphorylating RyR2 to increase open probability and calcium release (Meissner, 2004; Ai et al., 2005; Currie, 2009). The potential to directly phosphorylate Cav1.x channels, IK channels, or JPH3/4 in this process are all possible outcomes of CaMKII actions that could identify final effectors of delayed facilitation important to generating the sAHP.

## Factors Contributing to Slow Afterhyperpolarization Properties

Studies on the sAHP were met with continual frustration given a lack of pharmacological tools that investigators rely on to define the underlying basis of a response. As laid out above, identifying an expression of the IK channel isoform and potential heteromerization with other members of the KCNN family will help resolve this (Turner et al., 2015; Higham et al., 2019). Another significant development was identifying the role of the Na-K pump that overlaps and greatly extends the earlier calcium-dependent component (Gulledge et al., 2013; Tiwari et al., 2018). The magnitude of the Na-K pump contribution is also highly dependent on the history of spike firing, complicating efforts to define sAHP duration using only potassium channel blockers (Tiwari et al., 2018). Apart from an unrecognized contribution by the Na-K pump, a review of the conditions and techniques used to study the sAHP reveal several practices that have differed between labs over time. While many of these have been mentioned within this review, a brief summary for future investigators to consider is provided here.

An obvious difference that will have effects on the sAHP is the recording temperature that is typically set at either room temperature (22°C) or ∼32–34°C. It is known that the kinetics of ion channels can be markedly affected by temperature, as can the activity of kinases that can be required for their function. Temperature is also particularly relevant to the Na-K pump in which an increase of only 2°C from 34 to 36°C was reported to substantially increase the contribution of this component compared to the earlier calcium-dependent component (Gulledge et al., 2013). On the one hand this effect could be used to advantage if restricting recordings to ∼32°C help isolate the calcium-dependent component for study. But any studies conducted at temperatures approaching 36°C can be expected to incorporate both processes involved in generating the sAHP and need to consider how this might affect the apparent kinetics of the sAHP.

Early studies that first defined the sAHP used microelectrodes, followed later by almost exclusive use of patch clamp recordings. For most of a decade little attention was paid to the ionic constituents of the internal medium for whole-cell patch recordings before adjusting to ensure a reasonable equilibrium potential for key ions. Whole-cell recordings are also now recognized to wash out some component(s) required for the Na-K pump, leading to a return to the use of microelectrodes to see the full impact of this factor on the sAHP and the effects of temperature and preceding spike patterns (Gulledge et al., 2013; Tiwari et al., 2018).

Another factor that came to be recognized is that of age, where several contributing factors such as Cav1 channel and RyR expression change rapidly during development or are elevated in aged animals (Campbell et al., 1996; Thibault and Landfield, 1996; Gant et al., 2006; Kim et al., 2007; Raza et al., 2007). Reports on the properties of the IsAHP conducted during the early years of whole-cell patch recordings must then consider the accepted practice at the time of preparing slices from animals ∼P14. A similar consideration clearly applies to recordings conducted in dissociated hippocampal cultures from cells at undetermined levels of very early development. Cell-to-cell variation in spike output properties has been reported depending on their position over the dorso-ventral axis of the hippocampus and thus different projection targets (Dougherty et al., 2012; Hönigsperger et al., 2015). Some of these differences could reflect the properties (amplitude, duration) of the sAHP found within these subpopulations. The pattern of spike output can in turn regulate the sAHP over a longer time frame, such as through potentiation of the sAHP by differential recruitment of RyRs (Borde et al., 1999; Tedoldi et al., 2020). It is worth noting that the rate of repeated current pulse injections capable of inducing LTP of the sAHP is surprisingly low (1/min) (Borde et al., 1995), which is well within the standard background repetition rate for many labs.

From the time of even some of the earliest studies, the sAHP was found to be remarkably sensitive to block by neurotransmitters that activate second messenger pathways, such that noradrenergic and muscarinic cholinergic agonists became standard pharmacological tools to study the sAHP (Madison and Nicoll, 1982, 1984; Lancaster and Nicoll, 1987). These studies were not summarized in the current review given the complexities of second messenger regulation, but an effort to summarize these findings is needed in the field. This is emphasized even more given that recent studies reveal that the Na-K pump component of the sAHP is also highly sensitive to several kinases and phosphatases (Mohan et al., 2019, 2021; Tiwari et al., 2019). This includes noradrenergic and cholinergic agonists, indicating the need to reevaluate the early results obtained using some of the only tools that were available to study the AHP and always interpreted in the context of calcium-dependent sAHP channels.

The hyperpolarizing influence of the Na-K pump at least partially overlaps the potassium-mediated component, so it is difficult to comment on factors that define the kinetics of the sAHP. However, the ability for both a Cav1-RyR-IK complex and a Na-K pump to generate an sAHP in the same cell provides an excellent example of the concept of degeneracy, defined as the ability for structurally different elements to provide an analogous function (Rathour and Narayanan, 2019; Goaillard and Marder, 2021). This process has become increasingly recognized as a means for a system to have alternate routes by which one can generate a functional result that could be subject to different modulatory influence (Tiwari et al., 2019). The debate that has existed on the role for SK or Kv7 channels in generating the mAHP in CA1 pyramidal cells likely reflects another example of degeneracy that emerges under different conditions to generate this response (Gu et al., 2005; Chen et al., 2014; Mateos-Aparicio et al., 2014). Finally, almost no consideration has been made of the potential for alternative calcium sources like IP3 or ligand-gated receptors, or STIM-ORAI interactions to potentially contribute to generating the sAHP that needs further consideration.

## Conclusion

Defining the molecular basis for the sAHP in CA1 pyramidal cells has been a gradual process that unfolded over the course of 30 years since its initial description, with thousands of studies defining its properties and functions at the cellular, circuit, and behavioral levels. Recent work on both calcium- and calcium-independent components that drive the sAHP have resolved numerous complexities in its pharmacology and raised ever more questions on the number of factors that could modulate its activation. Yet differences in reported results remain, indicating that the final answer to the molecular identity of sAHP channels is still an active topic for research. Given these advances it is safe to assume that many more studies will be enabled to fully understand how a response as important as the sAHP contributes to regulating cell excitability.

## Author Contributions

Both authors listed have made a substantial, direct, and intellectual contribution to the work, and approved it for publication.

## Conflict of Interest

The authors declare that the research was conducted in the absence of any commercial or financial relationships that could be construed as a potential conflict of interest.

## Publisher’s Note

All claims expressed in this article are solely those of the authors and do not necessarily represent those of their affiliated organizations, or those of the publisher, the editors and the reviewers. Any product that may be evaluated in this article, or claim that may be made by its manufacturer, is not guaranteed or endorsed by the publisher.
